# Two-Phase Lineage Specification of Telencephalon Progenitors Generated From Mouse Embryonic Stem Cells

**DOI:** 10.3389/fcell.2021.632381

**Published:** 2021-04-16

**Authors:** Makoto Nasu, Shigeyuki Esumi, Jun Hatakeyama, Nobuaki Tamamaki, Kenji Shimamura

**Affiliations:** ^1^Department of Health Sciences, Faculty of Life Sciences, Kumamoto University, Kumamoto, Japan; ^2^Department of Anatomy and Neurobiology, Graduate School of Medical Sciences, Kumamoto University, Kumamoto, Japan; ^3^Department of Brain Morphogenesis, Institute of Molecular Embryology and Genetics, Kumamoto University, Kumamoto, Japan; ^4^Department of Morphological Neural Science, Graduate School of Medical Sciences, Kumamoto University, Kumamoto, Japan

**Keywords:** telencephalon, patterning, D–V axis, progenitor specification, mouse ES cells

## Abstract

Proper brain development requires precisely controlled phases of stem cell proliferation, lineage specification, differentiation, and migration. Lineage specification depends partly on concentration gradients of chemical cues called morphogens. However, the rostral brain (telencephalon) expands prominently during embryonic development, dynamically altering local morphogen concentrations, and telencephalic subregional properties develop with a time lag. Here, we investigated how progenitor specification occurs under these spatiotemporally changing conditions using a three-dimensional *in vitro* differentiation model. We verified the critical contributions of three signaling factors for the lineage specification of subregional tissues in the telencephalon, ventralizing sonic hedgehog (Shh) and dorsalizing bone morphogenetic proteins (BMPs) and WNT proteins (WNTs). We observed that a short-lasting signal is sufficient to induce subregional progenitors and that the timing of signal exposure for efficient induction is specific to each lineage. Furthermore, early and late progenitors possess different Shh signal response capacities. This study reveals a novel developmental mechanism for telencephalon patterning that relies on the interplay of dose- and time-dependent signaling, including a time lag for specification and a temporal shift in cellular Shh sensitivity. This delayed fate choice through two-phase specification allows tissues with marked size expansion, such as the telencephalon, to compensate for the changing dynamics of morphogen signals.

## Introduction

The telencephalon is the most elaborate structure of the mammalian brain and is responsible for higher mental functions, such as memory, speech, value judgments, and sociality. These neural functions are achieved by complex neural networks composed of cells with distinct lineages and origins within the telencephalon. The telencephalon is roughly divided into three subregions along its dorsal–ventral (D–V) axis: (i) the dorsal midline tissues, including the hem, which appears to be lost during development; (ii) the dorsal telencephalon (pallium), mainly producing glutamatergic neurons and ultimately forming the cerebral cortex, and (iii) the ventral telencephalon (subpallium), which includes the ganglionic eminence (GE) producing mainly GABAergic neurons and the preoptic area (POA) producing GABAergic as well as cholinergic neurons (Shimamura et al., 1995; Wilson and Rubenstein, 2000; Rallu et al., 2002a; Schuurmans and Guillemot, 2002; Imayoshi et al., 2008; Medina and Abellán, 2009). The cerebral cortex is composed of cells originating both internally and externally. The main external source is the ventrally adjoining GE (Tamamaki et al., 1997; Métin et al., 2006; Wonders and Anderson, 2006). The GE is further subdivided into three parts: the rostrally located lateral and medial ganglionic eminences (LGE and MGE) and the caudal ganglionic eminence (CGE).

The development of these subregions must be precisely coordinated to achieve proper telencephalic organization. It is generally believed that secreted signaling molecules or morphogens form concentration gradients according to the spatial distance from their sources and that progenitors attain distinct identities according to the local signal intensity, termed the French flag model (Wolpert, 1969). The morphogen sonic hedgehog (Shh) and bone morphogenetic proteins (BMPs) determine the fate of the ventral and dorsal telencephalon, respectively (Marín and Rubenstein, 2001; Rallu et al., 2002a). Loss of Shh signaling in the telencephalon of Foxg1-driven Smoothened (Smo) conditional mutants, where the expression of Shh signal transducer, Smo, is missing in the whole telencephalon because of the telencephalon-specific expression of Cre recombinase in the Foxg1 locus, results in complete loss of the ventral properties (Fuccillo et al., 2004). On the other hand, the dorsal midline tissues are regulated by BMPs to form the cortical hem and choroid plexus (Hébert et al., 2002; Subramanian and Tole, 2009). Wnt proteins (WNTs) are another dorsalizing factor. Conditional loss of beta-catenin, a key mediator of canonical WNT signaling, results in loss of the dorsal telencephalon and expansion of the ventral telencephalon (Backman et al., 2005).

Recently, temporal patterning cues, in addition to spatial cues, have been recognized as critical for neural specification (Ribes and Briscoe, 2009; Rogers and Schier, 2011; Sagner and Briscoe, 2017). Patterning mechanisms may require signal dose dependence, time dependence, or both. Several *in vivo* studies have demonstrated that certain telencephalic subregional properties develop with a time lag. The expression of Nkx2-1, a marker for MGE/POA progenitors, is detected before that of Gsx2, a marker for LGE/CGE progenitors, in the ventral telencephalon. Moreover, swelling of the MGE precedes that of LGE by 1 or 2 days in mice (Corbin et al., 2000; Sousa and Fishell, 2010). The specification of dorsal midline tissues occurs before the embryonic day (E) 8.5, preceding the expression of Gsx2 at approximately E9.5 (Thomas and Dziadek, 1993). A simple morphogen concentration gradient does not well explain this temporal progression of telencephalic patterning. Two interpretations are possible. One is that the patterning of the telencephalon is achieved by a concurrent specification of multiple regions during an early phase, followed by delayed differentiation of Gsx2^+^ LGE progenitors during the later phase. The other is that each subregion in the telencephalon is specified within distinct time windows and that fate determination of Gsx2^+^ LGE progenitors occurs after Nkx2-1^+^ MGE progenitor induction. The latter scheme implies that the relevant fate determinant factor(s) act on MGE and LGE progenitors at different time points. This raises an intriguing question as to how the order of induction among subregions is determined. However, it is difficult to distinguish between these putative patterning mechanisms based on current experimental results *in vivo*. In addition, it is still unknown precisely when and for how long cells are capable of making fate choices. In the past decade, it has become possible to generate nascent telencephalon-like tissues (organoids) *in vitro* from mouse or human embryonic stem (ES) cells or induced pluripotent stem (iPS) cells using either a three-dimensional (3D) *in vitro* differentiation method or a two-dimensional (2D) method (Watanabe et al., 2005; Eiraku et al., 2008; Danjo et al., 2011; Cambray et al., 2012; Nasu et al., 2012, 2020b; Maroof et al., 2013; Arber et al., 2015; Tyson et al., 2015). *In vitro* studies have generally administered signaling cues for the entire duration of the developmental process under study, despite the potential advantages of *in vitro* experimental models for examining the temporal dependence of lineage specification signals compared with *in vivo* systems.

Here, we investigate the time-dependent specification of subregional progenitors in telencephalon using the 3D culture system, where subregional induction by morphogens can be achieved under controlled conditions. We provide evidence for time-dependent fate specification during development in which a short-lasting signal during a specific time window is sufficient to induce subregional progenitors; in other words, the timing rather than the duration of signal exposure is essential for the specification. Furthermore, the two ventral progenitors of the MGE/POA and LGE/CGE show different dose dependencies through a temporal shift in cellular responsiveness for Shh signaling. Taken together, the present study reveals novel developmental mechanisms for telencephalon patterning based on the interplay of dose and time dependence.

## Results

### Two Specific Time Windows for Ventral Fate Induction in the Telencephalon by Sonic Hedgehog Signaling

**FIGURE 1:**
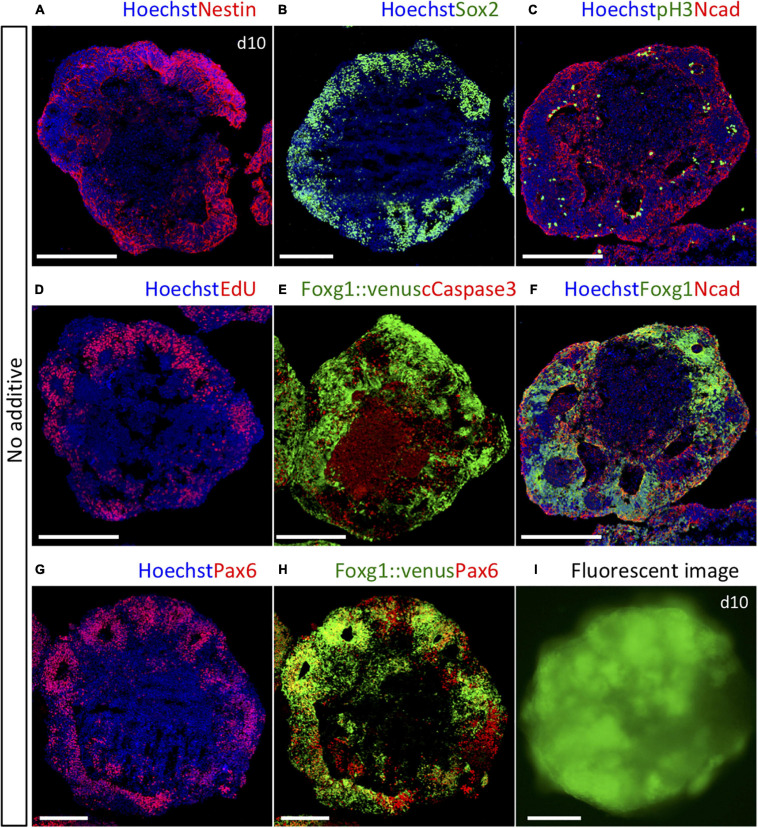
Foxg1::venus cell aggregates cultured in the default condition with no added cues. **(A)** Nestin^+^ neural lineage-committed cells were observed in most part of the outer zone. **(B)** Sox2^+^ neural progenitor cells were observed in the outer zone. **(C)** N-cadherin^+^ adherens junctions and pH3^+^ mitotic cells were located at the inner border of the outer zone. **(D)** EdU^+^ proliferating cells were dominantly located in the outer zone. **(E)** Cleaved caspase-3^+^ apoptotic cells were mostly retained in the inner cavity. **(F–H)** Majority of cells co-expressed Foxg1 and Pax6, indicating cortical fate. **(I)** Whole-mount fluorescence image of a Foxg1::venus aggregate. All aggregates were analyzed on day 10. Hoechst 33342 (blue), nuclear staining. Scale bars, 200 μm.

To identify essential temporal signaling processes for patterning the telencephalon, we used a 3D culture system to investigate how the subregional lineages of the telencephalon progenitors generated from mouse ES cells were specified in a temporal manner. The day of plating was defined as day 0. First, we verified proper neural induction using our method to inhibit transforming growth factor-beta (TGFb) signal during days 0–5 and WNT signal during days 1–5. The expressions of Nestin, a marker for neural lineage-committed cells, and Sox2, a neural progenitor cell marker, were observed in most parts of the outer zone of each aggregate (Figures 1A,B and Supplementary Figures 1A,B). N-cadherin, a marker for adherens junctions located at the apical side of the neuroepithelium, was detected at its inner border, whereas pH3, a mitotic cell marker, was located inside along the border (Figure 1C and Supplementary Figure 1E). EdU-labeled proliferating cells during days 9 and 10 were widely accumulated but exclusively in the outer zone (Figure 1D), indicating that our method effectively induced neural lineage cells, and neurogenesis occurred in the outer zone with the apical inside polarity, which is consistent with that reported by our previous study (Nasu et al., 2012). We observed that apoptotic cells were mostly retained in the inner cavity, detected by cleaved caspase-3, the active form of caspase-3 (Figure 1E). Induction of four lineage fates was immunocytochemically distinguished in developing telencephalic organoids by the following marker expression patterns on day 10 *in vitro*: Pax6^+^/Foxg1^+^ for the dorsal telencephalon (cortex), Gsx2^+^/Foxg1^+^ for the dorsal half of the ventral telencephalon (LGE/CGE), Nkx2-1^+^/Foxg1^+^ for the ventral-most telencephalon (MGE/POA), and Lmx1a^+^/Foxg1^–^ for the dorsal midline tissues (Supplementary Figures 1A–D,F,I). These four lineages were chosen because they emerge in a specific arrangement along with the D–V axis of the telencephalon *in vivo*, and both the location and temporal sequence of emergence are well documented (Toresson et al., 2000; Yun et al., 2001; Rallu et al., 2002a, b; Schuurmans and Guillemot, 2002; Corbin et al., 2003; Nasu et al., 2012). Using Foxg1::venus ES cells expressing fluorescent Venus specifically in telencephalic cells, we found that most neural lineage cells adopted the dorsal telencephalic progenitors, defined by co-expressing Foxg1 and Pax6, in the default condition with no added cues (Figures 1E–I).

**FIGURE 2:**
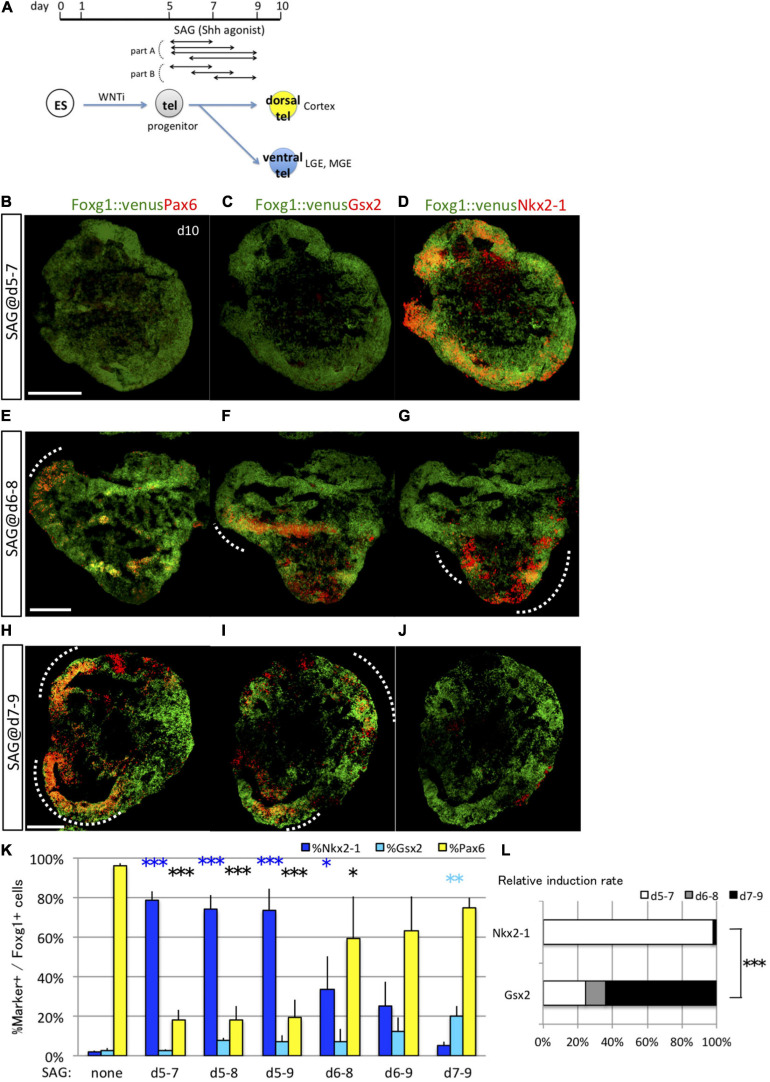
Analyses of the optimal Shh signal time window for induction of ventral fates in telencephalic organoids. **(A)** Schematic of the differentiation strategy for dorsal and ventral telencephalon (tel) using the 3D culture model. Embryonic stem (ES) cells can be differentiated into lineages of the anterior telencephalon by Wnt inhibition (WNTi; anteriorization) and lineages of the ventral telencephalon by Shh signaling (ventralization). We determined the optimal time window of Shh signal exposure to induce ventral telencephalon using an Shh agonist (SAG). Cell aggregates (organoids) were analyzed to express the pan-telencephalic marker Foxg1 and subregional markers on day 10. **(B–J)** Fluorescence images of serial sections through Foxg1::venus (telencephalic) cell aggregates (green) co-immunostained for subregional markers (red) Pax6 **(B,E,H)**, Gsx2 **(C,F,I)**, and Nkx2-1 **(D,G,J)**. A dashed line indicates an area with dual Foxg1^+^/subregional marker^+^ cells. Scale bars, 200 μm. **(B–D)** Aggregates cultured with 3-nM SAG during days 5–7 post-seeding expressed Nkx2-1 primarily. **(E–G)** Aggregates cultured with 3-nM SAG during days 6–8 expressed Pax6, Gsx2, and Nkx2-1. **(H–J)** Aggregates cultured with 3-nM SAG during days 7–9 expressed Pax6 and Gsx2 but little Nkx2-1. **(K)** Proportions (%) of subregional marker^+^ cells among Foxg1^+^ cells; Pax6^+^ (yellow bars), Gsx2^+^ (cyan bars), and Nkx2-1^+^ (blue bars). “d5–7,” “d6–8,” “d7–9,” “d5–8,” “d6–9,” and “d5–9” indicate SAG exposure during days 5–7, 6–8, 7–9, 5–8, 6–9, and 5–9, respectively. “None” indicates the culture condition without SAG. **(L)** Differences in relative induction rate during days 5–7 (white bars), 6–8 (gray bars), and 7–9 (black bars) indicate that the fates of Nkx2-1^+^ and Gsx2^+^ cells are determined at different times, with the early time window optimal for inducing Nkx2-1^+^ cells and the late time window optimal for inducing Gsx2^+^ cells. Values expressed as mean ± standard error of the mean (*N* = 3). ^∗^*P* < 0.05, ^∗∗^*P* < 0.01, ^∗∗∗^*P* < 0.001.

To investigate how long specific ES cell populations are sensitive to fate determination by signaling cues, we first focused on the known ventral fate determinant Shh. We utilized the advantage of chemical compounds to recombinant proteins, including high induction potency of signaling pathways, good stability in cell culture conditions, and less difference between manufacturing lots, to achieve tight control of the signal exposure (Chen et al., 2009; Danjo et al., 2011; Naujok et al., 2014). We examined its ventralizing effects of different durations of transient exposure to the Shh agonist SAG (3 nM) following the neural induction in the *in vitro* differentiation system (part A in Figure 2A). Exposure to SAG on day 5 resulted in increased ventral marker expression (Nkx2-1^+^) concomitant with decreased dorsal marker expression (Pax6^+^) among the Foxg1^+^ domain on day 10 (Figures 2B–D). The efficiency for general telencephalon induction (Foxg1::venus^+^ cells) was not influenced by the timing of SAG addition, indicating that telencephalic fate was maintained but ventralized by Shh signaling (Supplementary Figures 2A–G). Exposure on day 5 was the most effective condition for ventral marker induction, and similar numbers of Nkx2-1^+^ cells were induced by SAG exposure starting on day 5 independent of exposure duration (2–4 days) (days 5–7, 78.91 ± 4.50%; days 5–8, 73.86 ± 7.09%; days 5–9, 73.71 ± 10.80%) (Supplementary Figures 3A–F and Figure 2K). In contrast, induction of Nkx2-1^+^ cells (ventralization) was markedly reduced when 3 day SAG was administered starting on day 6 (day 6–9, 24.89 ± 12.24%) (Supplementary Figures 3G–I). Thus, SAG exposure for 2 days was sufficient to induce Nkx2-1^+^ (MGE/POA progenitor) cells. The critical condition for Nkx2-1^+^ cell induction is the specific timing of SAG application rather than the duration of SAG exposure.

Furthermore, Shh signaling specified distinct ventral lineage pathways depending on the timing of induction (part B in Figure 2A) in that the time window for the optimal induction of ventral-most MGE/POA (Nkx2-1^+^/Foxg1^+^) cells differed from that required for LGE/CGE (Gsx2^+^/Foxg1^+^) cell induction (Figures 2B–K). Although Nkx2-1^+^/Foxg1^+^ cells were efficiently induced by 2 day SAG exposure starting on day 5 but not on day 6 or 7 (days 6–8, 33.65 ± 16.92%; days 7–9, 5.01 ± 2.15%) (Figures 2D,G,J), Gsx2^+^/Foxg1^+^ cells were induced efficiently by 2 day SAG exposure starting on day 7 but poorly by exposure starting on day 5 or 6 (days 5–7, 2.85 ± 0.60%; days 6–8, 7.18 ± 6.11%; days 7–9, 20.12 ± 4.74%) (Figures 2C,F,I). Longer SAG exposure also slightly increased Gsx2^+^/Foxg1^+^ cell numbers (days 5–8, 7.90 ± 1.00%; days 5–9, 7.06 ± 3.39%; days 6–9, 12.19 ± 7.25%), but induction was clearly more sensitive to the timing of SAG exposure than the duration of exposure.

The induction rate of Pax6^+^ cells was inversely related to the induction rate of Nkx2-1^+^ cells, as numerous Pax6^+^ cells were induced with and without SAG exposure from days 7–9 (Figures 2B,E,H). SAG addition on day 6 resulted in a mixture of the three progenitors (Figures 2E–G and Supplementary Figures 3G–I). The relative induction rates differed significantly among cultures exposed to SAG for 2 days but starting on day 5, 6, or 7 (Figure 2L). Collectively, these results revealed two distinct time windows for the specification of ventral telencephalic progenitors by Shh, days 5–7 for Nkx2-1^+^ MGE/POA progenitors and days 7–9 for Gsx2^+^ LGE/CGE progenitors.

### Lateral Ganglionic Eminence/Caudal Ganglionic Eminence-Like Post-mitotic Neurons Were Produced by Late Exposure to the Sonic Hedgehog Agonist

**FIGURE 3:**
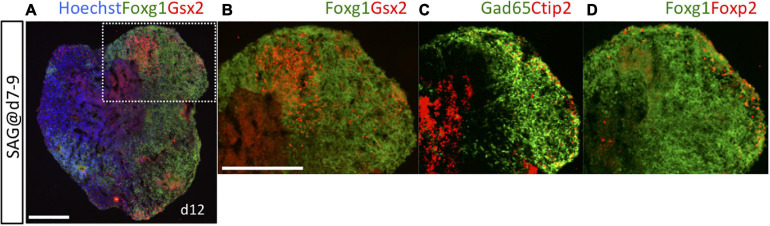
Post-mitotic LGE neurons were detected on day 12. Cell aggregates were cultured with 3-nM SAG during days 7–9 and analyzed on day 12. **(A)** Fluorescence image of an entirely Foxg1::venus^+^ aggregate (green) immunostained for Gsx2 (red) to show the induction of LGE progenitors. Hoechst 33342 (blue), nuclear staining. White dotted rectangular area is magnified in **(B–D)**. **(B)** Gsx2^+^/Foxg1^+^ LGE progenitors are located at the inner side within an area of Foxg1^+^ cells. **(C)** Expression of the pan-GE post-mitotic neuron markers Gad65 and Ctip2. **(D)** Expression of the LGE-specific post-mitotic neuron marker Foxp2. Foxp2^+^ neurons and Ctip2^+^ neurons are localized in the marginal areas of Gad65^+^ and Foxg1^+^ zones. Scale bars, 200 μm.

Gsx2 is expressed mainly in LGE/CGE progenitor cells *in vivo* and also at low levels in a small number of MGE progenitors (Supplementary Figure 1F). To verify that the Gsx2^+^/Foxg1^+^ cells analyzed on day 10 were indeed LGE/CGE progenitors, we characterized neurons generated from these progenitors by extending the culture period up to the emergence of post-mitotic neurons (day 12). Under SAG exposure during days 7–9 (optimal for Gsx2^+^/Foxg1^+^ induction over Nkx2-1^+^/Foxg1^+^ induction), Gsx2^+^/Foxg1^+^ LGE/CGE progenitor cells were observed at the luminal side of organoids (Figures 3A,B), which corresponds to the apical side of the brain wall *in vivo* (Nasu et al., 2012). Post-mitotic neurons were located in the marginal zone and specifically labeled with the pan-GE neuronal markers Gad65 and Ctip2 (Figure 3C and Supplementary Figures 1G,H) and the LGE/CGE-specific neuronal marker Foxp2 (Figure 3D and Supplementary Figures 1F,G). Gad65^+^ cells, which include both post-mitotic neurons and intermediate progenitors (Wu et al., 2011), were more widely distributed than were Ctip2^+^ or Foxp2^+^ cells. No Foxp2^+^ cells were observed in the vicinity of Nkx2-1^+^/Foxg1^+^ MGE/POA progenitor cells under SAG exposure during days 5–7 (not shown). It is thus reasonable to assume that these Gsx2^+^ progenitors produced post-mitotic neurons in the LGE/CGE. Furthermore, *in vitro*-generated Gsx2^+^ or Nkx2-1^+^ progenitors appear to adequately mimic lineage fate *in vivo*.

### Sonic Hedgehog Dose-Dependent Induction of Nkx2-1 and Dose-Independent Induction of Gsx2

**FIGURE 4:**
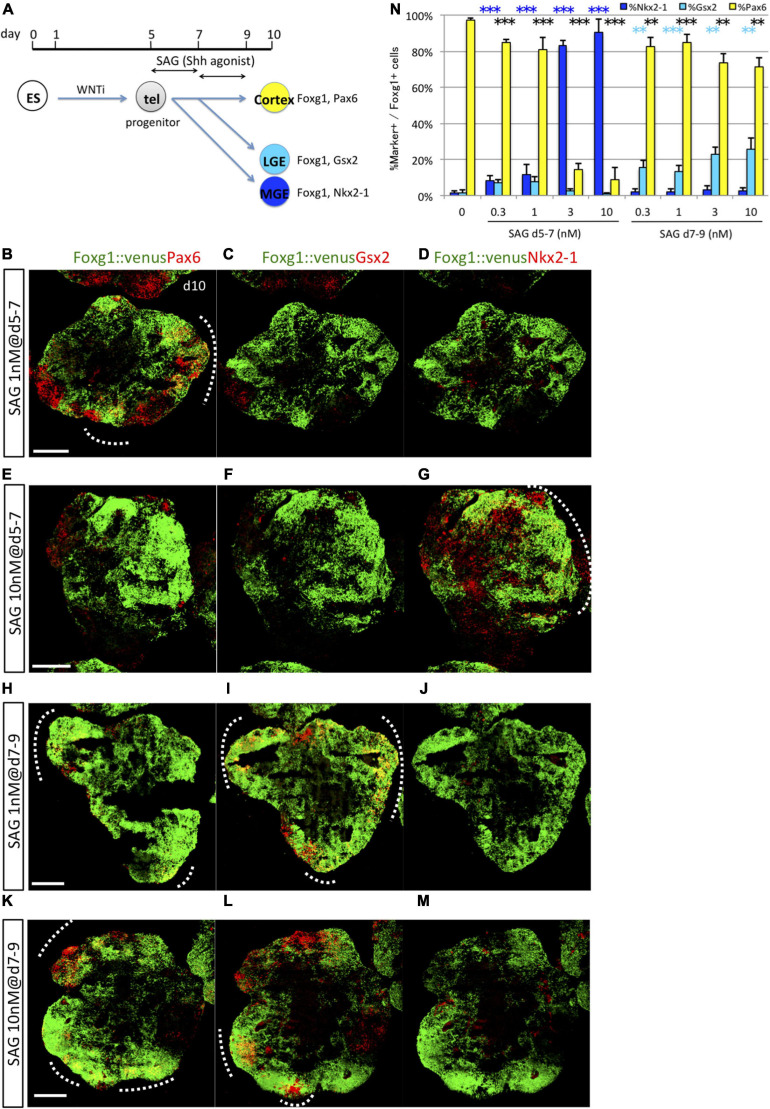
Dose-dependent induction of Nkx2-1 and dose-independent induction of Gsx2. **(A)** Schematic of the 3D culture used to investigate dose-dependent subregional marker expression in two time windows, days 5–7 and 7–9. **(B–M)** Fluorescence images of serial sections through Foxg1::venus (telencephalic) cell aggregates (green) co-immunostained for subregional markers (red) Pax6 **(B,E,H,K)**, Gsx2 **(C,F,I,L)**, and Nkx2-1 **(D,G,J,M)**. A dashed line indicates an area with dual Foxg1^+^/subregional marker^+^ cells. Scale bars, 200 μm. **(B–D)** Aggregates cultured with 1-nM SAG during days 5–7 post-seeding expressed Pax6 primarily. **(E–G)** Aggregates cultured with 10-nM SAG during days 5–7 expressed Nkx2-1 primarily. **(H–M)** Aggregates cultured with 1-nM (**H–J**) and 10-nM (**K–M**) SAG during days 7–9 expressed Pax6 and Gsx2 but little Nkx2-1. **(N)** Proportion (%) of subregional marker^+^ cells among Foxg1^+^ cells; Pax6^+^ (yellow bars), Gsx2^+^ (cyan bars), and Nkx2-1^+^ (blue bars). SAG was added to the culture during days 5–7 or 7–9 at the following concentrations: 0 (none), 0.3, 1, 3, and 10 nM. Results expressed as mean ± SEM (*N* = 4). ^∗∗^*P* < 0.01, ^∗∗∗^*P* < 0.001.

The present study revealed that specification of MGE/POA (Nkx2-1^+^/Foxg1^+^) progenitors and LGE/CGE (Gsx2^+^/Foxg1^+^) progenitors by Shh is dependent on the precise timing of first exposure (starting on days 5 and 7, respectively) but is not markedly influenced by absolute duration of Shh exposure. On the contrary, previous studies reported that the ventral properties of the telencephalon were specified by Shh in a dose-dependent manner, such that MGE/POA cells were induced by a high concentration and LGE/CGE cells by a low concentration (Danjo et al., 2011). Thus, both dose-dependent and time-dependent mechanisms may be important for ventral specification, although this issue has not been investigated under conditions in which these contributions can be clearly distinguished. To clarify this issue, we tested different concentrations of SAG, ranging from 0.3 to 10 nM, during the early (days 5–7) and late (days 7–9) time windows (Figure 4A). During the early time window (days 5–7), Nkx2-1^+^ and Pax6^+^ cell induction rates showed reciprocal SAG dose dependencies (Figures 4B–N and Supplementary Figures 4A–L). Nkx2-1^+^ cells were efficiently induced by relatively high doses of SAG (82.96 ± 3.09% at 3 nM and 90.32 ± 7.52% at 10 nM) but sparsely by low doses (8.48 ± 2.62% at 0.3 nM and 11.66 ± 5.48% at 1 nM). Alternatively, Pax6^+^ cells were substantially differentiated under low-dose SAG (or no SAG) but poorly induced by high-dose Shh. These results indicate that Nkx2-1^+^ and Pax6^+^ lineages are positively and negatively regulated by Shh signaling, respectively, in a dose-dependent manner during the early time window. In contrast to MGE/POA and cortical cells, Gsx2^+^ cells were not efficiently induced by any dose of SAG during the early time window (below 7.7%) but were induced during the second time window by all SAG doses (15.32 ± 4.32% at 0.3 nM, 13.11 ± 3.86% at 1 nM, 22.98 ± 3.73% at 3 nM, and 25.89 ± 5.90% at 10 nM). Notably, Nkx2-1^+^ cells were not induced during the second time window, even at high doses of SAG (below 3.2%). In summary, Nkx2-1^+^ MGE/POA induction required both time- and dose-dependent Shh signaling mechanisms, whereas Gsx2^+^ LGE/CGE induction was also highly dependent on the timing of the Shh signal (in a later time window) but much less sensitive to Shh dose. The efficiency for the general induction of telencephalic progenitors (Foxg1::venus^+^ cells) was not substantially influenced by SAG concentration during either time window (Supplementary Figures 2H–P).

### Dorsal Midline Tissues Were Induced by Timed Bone Morphogenetic Protein and WNT Exposure

**FIGURE 5:**
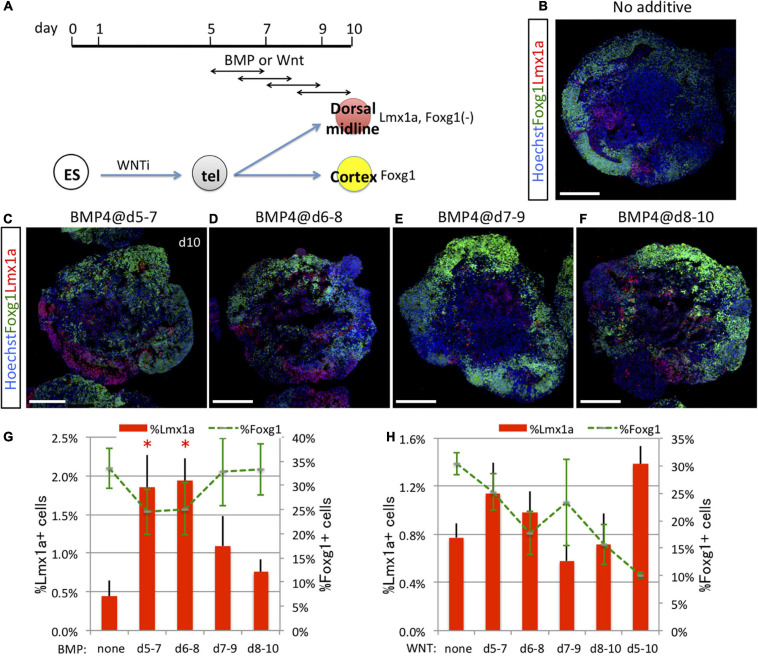
Dorsal midline tissues were induced by timed BMP and WNT exposure. **(A)** Schematic of differentiation strategy for dorsal midline tissues and cortex. ES cells differentiated into Pax6^+^/Foxg1^+^ cells (cortex) under the default condition (no additional cues) or into Lmx1a^+^ dorsal midline cells by BMP or WNT signaling (dorsalization). **(B–F)** Using the 3D culture, we assessed the optimal time window for 2 days of BMP signal (BMP4 recombinant protein) exposure to induce dorsal midline tissues. Foxg1::venus cell aggregates cultured without BMP4 **(B)** or with BMP4 during days 5–7 **(C)**, 6–8 **(D)**, 7–9 **(E)**, or 8–10 **(F)**. Images show Foxg1::venus fluorescence (green) and immunostaining for Lmx1a (red). Lmx1a^+^ cells (red) were induced in restricted patches but in contiguous arrangement with Foxg1^+^ cortical cells (green) as *in vivo*. Hoechst 33342 (blue), nuclear staining. Scale bars, 200 μm. **(G)** Proportion (%) of subregional marker^+^ cells among the whole aggregate; Lmx1a^+^ (red bars) and Foxg1^+^ (green dashed line). **(H)** We analyzed the optimal time window for 2 or 5 days of WNT signal exposure to induce dorsal midline tissues using the WNT agonist CHIR99021. Proportion (%) of subregional marker^+^ cells in aggregates; Lmx1a^+^ (red bars) and Foxg1^+^ (green dashed line). Expressed as mean ± SEM (*N* = 3). ^∗^*P* < 0.05.

The pallium (cortex) is in contact with the subpallium (the MGE in early development) at the pallial–subpallial boundary and with dorsal midline tissues at the hem–cortex boundary (HCB). Results presented in Figures 1, 2 suggest that pallial vs. subpallial fate at the pallial–subpallial boundary is determined by Shh signaling during a specific temporal window (days 5–7 *in vitro*). To address when the fate of hem vs. cortex progenitors at the HCB is induced by a dorsalizing signal, we focused on BMP and WNT, which are known signaling factors for dorsalization in the telencephalon and for fate choice at the HCB between dorsal midline and cortical tissues (Eiraku et al., 2008; Watanabe et al., 2012). We first examined the time window for efficient induction of Lmx1a^+^ dorsal midline tissue by BMP signaling (exposure to recombinant BMP4) (Figure 5A). Similar to that in the case *in vivo*, where the Lmx1a^+^ hem adjoins the dorsal Foxg1^+^ cortex at the HCB within the Sox2^+^ neuroepithelial sheet (Supplementary Figure 1A), *in vitro*-generated Lmx1a^+^ cells were frequently adjacent to Foxg1::venus^+^ cells independent of induction efficiency (Figures 5B–F). Consistent with a previous report (Watanabe et al., 2012), early BMP signaling starting on day 5 or 6 (but not day 7 or 8) efficiently induced Lmx1a^+^ cells at the cost of Foxg1::venus^+^ cells (days 5–7, 1.85 ± 0.42%; days 6–8, 1.93 ± 0.29%; days 7–9, 1.09 ± 0.39%; days 8–10, 0.76 ± 0.16%, no BMP, 0.44 ± 0.21%; expressed as a percentage of all Foxg1::venus^+^ cells in aggregates) (Figure 5G), indicating that BMP is a key signal for the fate choice between midline and cortical lineages at the HCB *in vitro*. Next, we examined the time window for efficient induction of Lmx1a^+^ dorsal midline tissue by WNT signaling using the Wnt agonist CHIR99021 (Figure 5A). WNT signaling alone did not markedly induce Lmx1a^+^ cells, although numbers were numerically higher with stimulation starting on day 5 (days 5–7 or 5–10) compared with day 7 or 8 (Figure 5H). WNT signal had weaker dorsalizing effects than did BMP signal; however, WNT-containing conditions were strongly negative for Foxg1 expression (Figure 5H), which contrasts with the modest decrease of Foxg1 by BMP signal (Figure 5G). Earlier or longer WNT exposure resulted in fewer Foxg1^+^ cells, possibly because of the caudalizing effects of the WNT signal.

**FIGURE 6:**
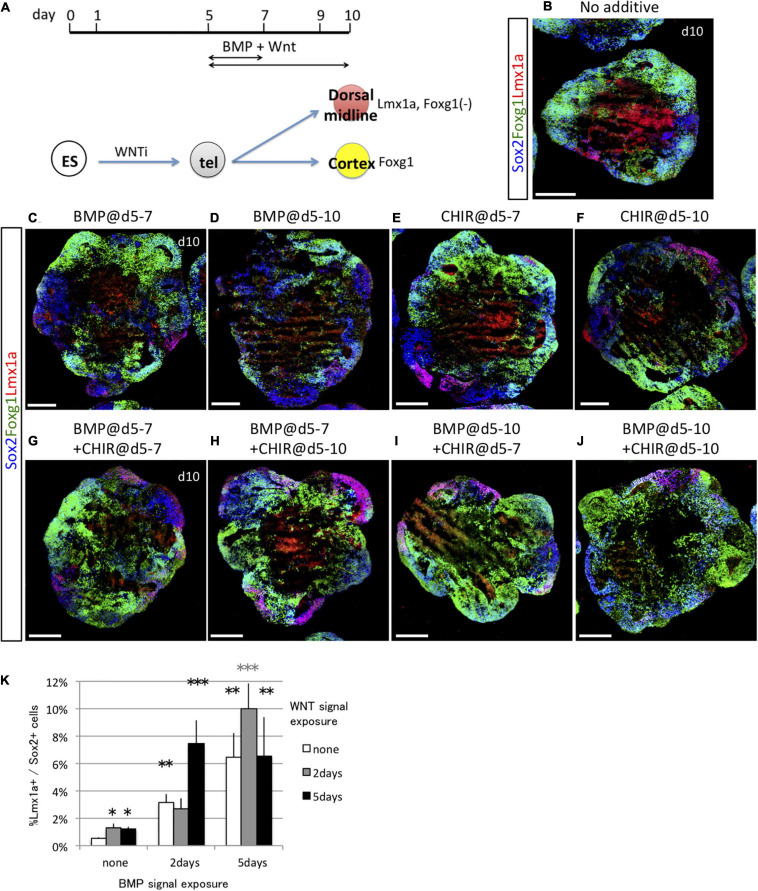
Dorsal midline tissues were induced by timed BMP and WNT exposure. **(A)** Schematic of differentiation strategy for dorsal midline tissues and cortex. **(B–F)** Using the 3D culture, we assessed the optimal time window for 2 days (days 5–7) or 5 days (days 5–10) of BMP signal (BMP4 recombinant protein) or WNT signal (CHIR) exposure to induce dorsal midline tissues. Foxg1::venus cell aggregates cultured with no cues **(B)** or with BMP4 during days 5–7 and days 5–10 **(C,D)**, with CHIR during days 5–7 and 5–10 **(E,F)**. Images show Foxg1::venus fluorescence (green) and immunostaining for Lmx1a (red) and Sox2 (blue). **(G–J)** We examined the synergistic effect of BMP and WNT signals in combination for 2 days (days 5–7) or 5 days (days 5–10). Scale bars, 200 μm. **(K)** Proportion (%) of Lmx1a^+^ cells among Sox2^+^ cells. Greater proportion (%) of Lmx1a^+^ cells among Sox2^+^ cells indicates that WNT signaling enhances the dorsalizing effect of BMP. Expressed as mean ± SEM (*N* = 3). *P < 0.05, **P < 0.01, ***P < 0.001.

Subsequently, we investigated whether BMP and WNT signals acted synergistically to induce the dorsal midline fate (Lmx1a^+^) when present for 2 or 5 days starting on day 5 (Figure 6A). Foxg1 expression was negatively regulated by both BMP and WNT, whereas WNT had a greater negative effect on Foxg1 expression (Supplementary Figures 5A,B). To focus on the fate determination of the dorsal midline vs. cortical cells, this analysis was limited to Sox1^+^ neural progenitor cells because both Foxg1^+^ cells and Lmx1a^+^ cells co-expressed Sox2 (Figures 6B–J). Similar to the ventralizing signal, a short period of BMP4 and/or CHIR99021 exposure (2 days) was of Lmx1a^+^/Sox2^+^ dorsal midline cells, although a longer exposure (5 days) might be more effective (Figure 6K). BMP and WNT synergistically induced more than 10% of dorsal midline cells. Thus, the fates of cortical cells and dorsal midline tissues were determined by both BMP and WNT signals, mainly during days 5–8. Collectively, cortical fate was negatively influenced by a dorsalizing and a ventralizing factor during the same period.

### Gsx2^+^ Lateral Ganglionic Eminence/Caudal Ganglionic Eminence Induction Required Subthreshold Levels of Sonic Hedgehog and Wnt Signal During the Early Phase

The fates of the two ventral subregions, MGE/POA and LGE/CGE, were determined through a two-phase specification process dependent on distinct mechanisms; early progenitors produced ventral cells (MGE/POA) through strongly dose-dependent Shh signaling, whereas late progenitors required Shh signaling for ventral specification (LGE/CGE) but with much lower dose sensitivity. Importantly, early progenitors had little competence for differentiating into Gsx2^+^ cells and late progenitors for differentiating into Nkx2-1^+^ cells, implying that the Shh signaling response shifts from the early to late phase. For the induction of the LGE/CGE fate, progenitors must first remain resistant to both ventralizing and dorsalizing signals during early development to avoid the ventral-most (MGE/POA) and the dorsal (cortex) fate and must then respond to a later Shh signal.

**FIGURE 7:**
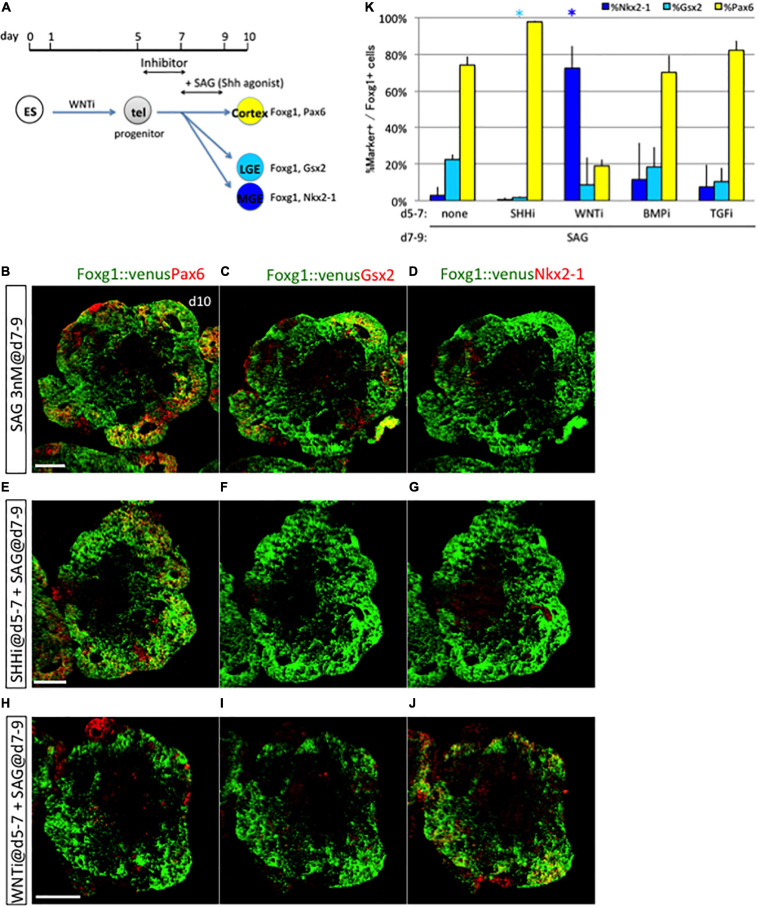
Prerequisite condition for subsequent LGE/CGE induction. **(A)** Schematic of the 3D culture used to identify the signaling pathway responsible for LGE/CGE induction. Disruption of LGE/CGE induction by Shh signal exposure during days 7–9 due to the absence of any endogenous signal during days 5–7 indicates the need for a prerequisite condition. Inhibitors for Shh signal (cyclopamine), WNT signal (WNTi), BMP signal (LDN193189), and TGFb signal (SB 431542) were added to the culture during days 5–7 with subsequent Shh signal exposure (SAG) during days 7–9. **(B–J)** Aggregates cultured without any inhibitor **(B–D)** or with inhibitors for the SHH **(E–G)** and WNT **(H–J)** signaling pathway during days 5–7 and then treated with SAG during days 7–9. Scale bars, 200 μm. **(K)** Proportion (%) of subregional marker^+^ cells among Foxg1^+^ cells; Pax6^+^ (yellow bars), Gsx2^+^ (cyan bars), and Nkx2−1^+^ (blue bars). Early treatment with Shh and WNT signaling inhibitors reduced Gsx2^+^ cell induction by later SAG exposure. Results expressed as mean ± SEM (*N* = 3). *P < 0.05.

Some endogenous signals may be responsible for this shift in specification competency (Sasai et al., 2014). We thus examined how progenitors change signal dependence *in vitro*. To clarify whether an endogenous early signal is involved, we examined expression dynamics of the above D–V patterning factors—Wnt3a, BMP4, and Shh—and Tgfb1 as a reference from an ES cell maintenance state (day 0) through neural differentiation using quantitative polymerase chain reaction (qPCR). Consistent with the previous study, we detected quite low but certain expressions of Wnt3a, Shh, and Tgfb1 and a high expression of Bmp4 in ES cells (day 0), where BMP4 signaling might be distinct from differentiation stages (Supplementary Figures 6A–D; Bertacchi et al., 2015; Morikawa et al., 2016). We confirmed that those expressions were sustained and dynamically changed in the course of neural differentiation. Next, we examined inhibiting the activity of selective inhibitors of Shh, WNT, BMP, and TGFb signaling pathway (termed SHHi, WNTi, BMPi, and TGFi, respectively) during the early phase of culture under baseline conditions (i.e., no Shh, BMP, or Wnt agonists). SHHi significantly downregulated its downstream target, Gli1, but not Patched 1 (Ptc1) (Supplementary Figures 6E,F). WNTi efficiently reduced the amount of beta-catenin, a downstream target in the WNT pathway (Supplementary Figures 6H,K). Although BMPi and TGFi slightly but not significantly reduced the number of phosphorylated Smad1/5 (pSmad1/5) and phosphorylated Smad2 (pSmad2), a downstream target in the BMP pathway and the TGFb pathway, respectively (Supplementary Figures 6I,J,L,M), it might be partially due to the activity of BMP4 and TGFb signal transduction that was still low under baseline conditions (Supplementary Figures 6B,D). Then, we applied SHHi, WNTi, BMPi, and TGFi during the early phase, followed by late exposure to the Shh agonist (Figure 7A). Both SHHi and WNTi treatment, but not BMPi and TGFi treatment, during days 5–7 dramatically reduced Gsx2 + cell induction in response to late Shh agonist exposure and modified fate determination (Figures 7B–K and Supplementary Figures 7A–F). SHHi basically induced the cortical fate, and WNTi drastically increased Nkx2-1^+^ cell numbers. These findings indicated that both Shh and WNT signals during days 5–7 are a prerequisite for later LGE/CGE induction by Shh. Given that early Shh and WNT signals are induction factors for ventral-most (MGE/POA) and dorsal midline tissues, respectively, they must be at subthreshold levels to evoke the temporal shift in specification competency. Gene expression state of environmental factors, such as Shh, Wnt3a, and Bmp4, but not Tgfb1, were different between days 5 and 7, which should be derived from their own cell aggregates (Supplementary Figures 6A–D). Two target genes of the Shh signaling pathway, Gli1 and Ptc1, remained unchanged during days 5–7 (Supplementary Figures 6E,F), whereas Smoothened (Smo), one of the essential Shh signaling components, was downregulated from days 5 to 6 and 7 (Supplementary Figure 6G). These indicated that neuronal progenitors change their expressional profiles during their lineage commitment and temporally modify cellular responsiveness for the Shh signaling pathway and the environmental signaling state.

**FIGURE 8:**
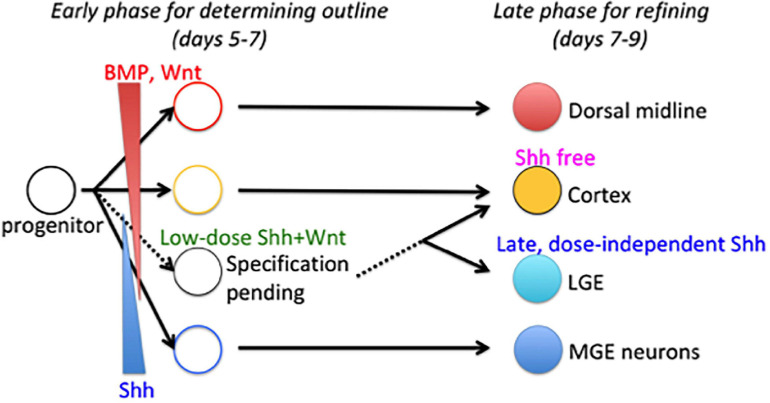
Two-phase specification model for telencephalon patterning. Fate of dorsal midline telencephalic cells is determined by BMP/WNT signaling, whereas the fate of ventral-most telencephalon (MGE) cells is determined by a high dose of Shh signal during the early phase (shown by solid lines). Fate determination of intermediate subregions is conditional (shown by dashed lines). LGE is specified by a dose-independent Shh signal during the late phase through a temporal shift in progenitor states for the Shh response.

Taken together, our data propose the two-phase specification model to explain the delayed specification of LGE/CGE progenitors relative to MGE/POA and cortical progenitors (Figure 8). The model postulates that whole tissue organization is achieved in two phases. In the early phase, the fates of subregions close to signal sources are determined by exposure to high morphogen concentrations (a dominant morphogen), whereas the fate of subregions receiving multiple signals remains pending. In the late phase, all the pending fates were determined based on temporal changes in environmental cues or progenitor competence. The present study demonstrates that the changes in signal dependence for progenitor specification are essential for fate determination during the late phase and are rendered by subthreshold Shh and WNT signals during the early phase.

## Discussion

In the present study, we highlight the concerning points and topics of telencephalon patterning. We verified the critical contributions of three key signaling factors for lineage specification of subregions in the telencephalon, ventralizing Shh and dorsalizing BMP and WNT. By applying these factors at different concentrations and time points, we achieved selective induction of subregional progenitors from mouse ES cells. According to the accepted patterning model based on positional information, the dose is a key determinant of morphogen response, such that tissues at short or long distances from a signal source are differentially induced or are both induced but by high and low doses of the signal, respectively (Wolpert, 1969; Campbell, 2003; Medina and Abellán, 2009). Our results appear consistent with this model in that cells were induced to express Nkx2-1 (MGE/POA lineage) by a high Shh dose, Gsx2 (LGE/CGE lineage) by a low Shh dose, and Lmx1a (the dorsal midline tissue lineage) by BMP and WNT. In addition, we also found that the timing of signal exposure for efficient induction was specific to each lineage; early exposure to Shh efficiently induces MGE/POA progenitors, whereas late exposure to Shh induces LGE/CGE progenitors, and early BMP/WNT signal exposure induces dorsal midline tissues. Cortical fate (Pax6^+^/Foxg1^+^) was a default (signal-independent) state in the early stage. These suggest that the fate of most telencephalic cells is determined concurrently, whereas that of a small group, the LGE/CGE progenitors, is delayed (for 2 days in mice). Our results provide evidence that both the appropriate dose and correct timing are critical for telencephalic patterning by morphogens.

The early patterning field may be so small that the middle domain receives multiple morphogens derived from distinct signal sources. Three possible mechanisms could allow cells within this domain to respond selectively or correctly interpret multiple patterning cues as a fate determinant: (i) one morphogen is the dominant fate determinant (i.e., due to a higher concentration or greater sensitivity of the cell), (ii) multiple morphogens work together (i.e., the cell requires the synergistic actions of multiple morphogens for fate determination), and (iii) fate determination is conditional (e.g., contingent on a prior priming signal). In the third case, cellular changes are required to prepare the cell for subsequent fate determination. Considering our results showing delayed specification of LGE/CGE progenitors relative to MGE/POA and cortical progenitors, the latter two-phase specification model is favored (Figure 8). The early phase determines an outline of the tissue subdivision, and the late phase refines the pattern.

Although morphogens continue to be produced throughout the expansion of the telencephalon, the size of the prospective patterning field changes up to several folds, and some distinct regional characteristics emerge progressively. When the patterning field expands with tissue growth, signal-receiving cells must decode the spatiotemporally changing status of signals. In addition to positional information underlying the classic model, temporal information may be a critical signaling domain of patterning cues for neural specification (Ribes and Briscoe, 2009). Several possible mechanisms could confer dose dependence, time dependence, or both to fate-determining signals (Sagner and Briscoe, 2017). (i) For dose dependence and time independence, progenitors could convert a transient graded signal into a sustained molecular change through induction of an autoregulatory feed-forward loop before extensive tissue growth. The specification should be contemporaneously completed within a relatively short period and before the onset of planar expansion of the neuroepithelial tissue. In this way, the progenitor could recognize the morphogen at an early stage as a pre-patterning signal (Moore et al., 2013). Such progenitors would exhibit robustness for the change in tissue size and a consequent change in signal dose. (ii) For time dependence and dose independence, the mechanism could involve sequential signal transduction or a signal-relay system in which signal recipient cells first react to the signal and then transfer it to other neighboring cells (van Boxtel et al., 2015). This sequential signal transduction would result in a temporal gradient of the signal. In this case, the timing of the input is the primary cue for fate determination, and signal transduction in the following cell population would be little affected by changes in absolute dose associated with tissue growth. (iii) Finally, a dose- and time-dependent mechanism is represented by two distinct cases. (iii-a) The integrated intensity of intracellular signal transduction could be important for some progenitors (Dessaud et al., 2007; Ribes and Briscoe, 2009). These progenitors would decode the cumulative amount of signal received during a certain period. In this case, the initial dose, the rate of change, and the exposure time would all be essential factors for establishing cell identity. When an ultrasensitive switch-like response to a morphogen signal occurs in an intracellular signaling pathway, a graded signal is converted to a transcriptional code, which is composed of Pax6, Gsx2, and Nkx2-1 in the telencephalon (Lek et al., 2010; Rogers and Schier, 2011; Shindo et al., 2016). When progenitors are exposed to subthreshold signals, they keep a graded response to the incremental signals, and graded induction will take time to be achieved (Zhang et al., 2006). In the case of (ii) and (iii-a), the signal duration is a key factor, and fate should differ depending on the length of signal exposure time. (iii-b) In the other case, to adopt the demand for a definite signal input with attenuation as the tissue size increases, progenitors may change competence for signal responsiveness as development proceeds (Rogers and Schier, 2011). A subthreshold signal or another signaling input may trigger the shift in signal dependence (Lek et al., 2010; Sasai et al., 2014; Srinivasan et al., 2014). In this case, recipient cells respond depending on their competence at that time and would be unaffected by both the initial dose and the rate of change. Our data demonstrate novel developmental mechanisms for telencephalon patterning based on both dose dependence in the early phase and time dependence, including a temporal shift in cellular Shh sensitivity, to induce ventral fate in response to Shh signaling. This delayed fate choice allows tissues with marked size expansion, such as the telencephalon or other tissues, to cope with the changing dynamics of patterning cues.

Conditions for the induction of Gsx2^+^ LGE/CGE had an adverse effect on Nkx2-1^+^ MGE/POA induction and *vice versa*, implying a repressive interaction between Nkx2-1 and Gsx2 or associated signaling pathways. It is compatible with previous findings in mutant mice (Rallu et al., 2002a, b; Schuurmans and Guillemot, 2002; Corbin et al., 2003). For instance, Shh^–/–^; Gli3^–/–^ double mutants and Nestin–Cre-driven Shh null mutants that lose Shh expression around E10–12 retained all telencephalic subdivisions but failed to express Nkx2-1 in the MGE and exhibit a concomitant expansion of Gsx2 expression into the MGE-like subdivision (Rallu et al., 2002b; Xu et al., 2005). Fate conversion of the MGE to LGE was also observed in Nkx2-1^–/–^ mutant mice (Sussel et al., 1999; Corbin et al., 2003), whereas mild mutants (Six3–Cre driving Smo mutants) exhibited a mosaic reduction of Nkx2-1 expression in the MGE with ectopic Gsx2 expression in the Nkx2-1-negative patches (Xu et al., 2010). Nkx2-1-Cre-mediated recombinant cells are not detected in LGE/CGE, indicating that Gsx2 and Nkx2-1 lineages are derived from different progenitors (Xu et al., 2008). The expression of Nkx2-1 comes before that of Gsx2 (Corbin et al., 2003). These findings strongly suggest that Nkx2-1 represses Gsx2, although it is currently unclear if this transcriptional regulation is direct. It also remains to be determined how presumptive LGE/CGE progenitors change competence in a time-dependent manner or how LGE/CGE progenitor specification is delayed relative to other telencephalic tissues. Possible explanations include slow accumulation of *de novo* synthesized proteins or degradation of existing proteins dependent on genetic or epigenetic regulation (Hirabayashi et al., 2009; Aldiri et al., 2017). The induction rate of LGE/CGE fate was still low under our conditions with a later phase of the Shh signal. Considering that the antibody used in this study did not detect the majority of MGE/POA progenitors, some of which expressed Gsx2 at a low level (Supplementary Figure 1F), it might fail to detect some LGE/CGE progenitors expressing Gsx2 at a low level, resulting in underestimation of the induction of LGE/CGE. Additional cues, such as FGF15/19 and Activin A, may be required for more effective induction of LGE/CGE fate (Danjo et al., 2011; Cambray et al., 2012). As MGE-derived neurons produce Shh (Nery et al., 2001; Sousa and Fishell, 2010), MGE-derived Shh may act as the subthreshold delay signal for subsequent LGE cell induction. Further studies are required to reveal the precise molecular signaling mechanisms underlying the delayed induction of LGE/CGE cells.

## Materials and Methods

### ES Cell Culture and Treatment With Soluble Factors

Mouse ES cells [Foxg1::venus (Eiraku et al., 2008)] were maintained as described previously (Watanabe et al., 2005). Briefly, ES cells were cultured on feeder-free, gelatin-coated dishes with a maintenance medium composed of Glasgow minimum essential medium supplemented with 10% Knockout Serum Replacement (Thermo Fisher Scientific), 1% fetal calf serum, 1 mM pyruvate (Sigma-Aldrich), 0.1 mM non-essential amino acids (Thermo Fisher Scientific), 0.1 mM 2-mercaptoethanol (Sigma-Aldrich), and leukemia inhibitory factor. The *in vitro* differentiation conditions for the 3D culture were as described in our previous study with minor modifications (Nasu et al., 2012). Briefly, ES cells were dissociated in 0.25% trypsin and quickly re-aggregated by plating on 96-well low cell-adhesion plates (Sumilon) in differentiation medium (5,000 cells per 100 μl/well). The differentiation medium was a growth factor-free chemically defined medium (gfCDM) composed of Iscove’s modified Dulbecco’s medium (Fujifilm Wako)/Ham’s F12 medium (Fujifilm Wako) 1:1, 1 × Chemically Defined Lipid Concentrate (Thermo Fisher Scientific), 450 μM monothioglycerol (Sigma-Aldrich), 5 mg/ml purified bovine serum albumin, 5.5 μg/ml apo-transferrin, 6.7 ng/ml selenium, and 10 μg/ml insulin. The day of ES cell seeding was defined as differentiation day 0. On day 5, cell aggregates were transferred to bacterial-grade, non-coated dishes with Dulbecco’s modified Eagle medium/Ham’s F12 medium supplemented with N2 and apo-transferrin (Fujifilm Wako). The TGF inhibitor SB 431542 (Cayman, 13031, IC50 = 94 nM) was added at a final concentration of 5 μM on day 0. The Wnt inhibitors IWP2 (Cayman, 13951, IC50 = 27 nM) and IWR1e (Cayman, 13659, IC50 = 180 nM) (WNTi) (Chen et al., 2009) were freshly prepared in 100 μl of gfCDM and added at final concentrations of 1 and 2 μM, respectively, on day 1. Matrigel (BD Biosciences) was prepared in 100 μl of gfCDM at a final concentration of 100 μg/ml and added on day 1. Human recombinant FGF8 (humanzyme, HZ-1104) was added at a final concentration of 50 ng/ml during days 5–7. The concentration, timing, and duration of SAG (Cayman, 11914, EC50 = 3 nM) exposure are described in the text and figures. The timing and duration of 1 ng/ml of BMP4 (humanzyme, HZ-1045) or 1 nM Wnt agonist CHIR99021 (Cayman, 13122, IC50 = 6.7 nM) exposure are described in the text and figures. The Shh antagonist cyclopamine (Cayman, 11321, IC50 = 24 nM) was added at 1 nM and the BMP inhibitor LDN193189 (Cayman, 11802, IC50 = 4.9 nM) at 10 nM on days 5–7 as indicated.

### Tissue Preparation

Jcl:ICR mice were killed at embryonic day 12 (E12) (purchased from CLEA Japan). All mice were anesthetized with medetomidine/midazolam/butorphanol tartrate/phosphate-buffered saline (PBS) (final dose, 0.3 mg/kg of body weight; 4 mg/kg of body weight; and 5 mg/kg of body weight, respectively) and perfused from the left ventricle with iced 4% paraformaldehyde (PFA)/PBS (pH 7.2). Extracted brain tissues were postfixed with 4% PFA/PBS (pH 7.2) for 1 h. Cell aggregates were harvested on day 10 or 12 and fixed with 4% PFA/PBS (pH 7.2) for 20 min. Fixed tissues and cell aggregates were cryoprotected in 15% sucrose/PBS overnight at 4°C and embedded in Tissue-Tek O.C.T. compound (Sakura Finetek). Frozen tissues were sliced at 12 μm. All animal experiments were performed in accordance with institutional (Kumamoto University) guidelines and were approved by the Animal Care and Use Committee of Kumamoto University.

### Immunostaining

Immunohistochemistry and immunocytochemistry were performed as described previously (Nasu et al., 2020a). Briefly, sections were rinsed with 0.3% Triton-X100/PBS three times and incubated overnight at 4°C with primary antibodies diluted in incubation buffer (5% donkey serum/0.3% Triton-X100/PBS) after pretreatment with incubation buffer for 30 min. The following primary antibodies were used at the indicated dilutions: anti-cCaspase3 (rabbit, 1:50; Cell signaling, 9664), anti-Ctip2 (rat monoclonal, 1:3,000; Abcam, ab18465), anti-Foxg1 (rabbit, 1:2,000; TaKaRa, M227), anti-Foxp2 (goat, 1:100; Santa Cruz, sc-21069), anti-Gad65 (mouse monoclonal, 1:200; BD Pharmingen, 559931), anti-Gsx2 (rabbit, 1:500; Millipore, ABN162), anti-Lmx1a (goat, 1:100; Santa Cruz, sc-54273), anti-N-cadherin (mouse, 1:1,000; BD Pharmingen, 610920), anti-Nestin (mouse, 1:200; BD Pharmingen, 611658/611659), anti-Nkx2-1 (mouse, 1:500; Leica, NCL-L-TTF-1), anti-Pax6 (rabbit, 1:2,000; BioLegend, PRB-278P), anti-pH3 (rabbit, 1:1,000; Millipore, 06-570), and anti-Sox2 (rabbit, 1:1,000; Millipore, AB5603). Hoechst 33342 was used for counterstaining of nuclei. Proliferating cells were labeled by EdU for 24 h from day 9 and detected chemically according to the manufacturer’s instructions (Thermo Fisher Scientific). Images were obtained using a BZ-X700 fluorescence microscope (Keyence) and laser-scanning confocal microscope (FV-1200, FV-1000, or FV-300; Olympus) and analyzed using BZ-X700 software and ImageJ. The percentage of Pax6^+^, Gsx2^+^, Nkx2-1^+^ fractions among Foxg1^+^ area was calculated. The expression of Pax6, Gsx2, and Nkx2-1 is based on nuclear staining, whereas the fluorescence of venus protein, corresponding to Foxg1 expression, localizes a whole cell. To revise underestimation caused by comparing nuclear staining fractions to a whole cell, the total percentage of three lineages was regarded as 100% because the telencephalon is subdivided by a transcriptional code, which is composed of Pax6, Gsx2, and Nkx2-1 (Rallu et al., 2002a, b; Schuurmans and Guillemot, 2002; Corbin et al., 2003). The percentage of Lmx1a^+^ and Foxg1^+^ positive fractions was calculated among whole aggregates (Figure 5) or among nuclear staining Sox2^+^ area (Figure 6). Five to eight aggregates from three or four biological replicates were analyzed.

### Quantitative Polymerase Chain Reaction

qPCR was performed using the ViiA7 Real-Time PCR System (Thermo Fisher Scientific). ES cells under maintenance culture (referred to as day 0) or cell aggregates under differentiation culture on days 3, 4, 5, 6, and 7 were harvested, and total RNAs were sampled for the qPCR study. Cell aggregates treated with SHHi during days 5–7 were harvested on days 6 and 7. Expression levels of target genes were obtained from duplicated, three biological replicates and shown as relative values, based on the expression of beta-actin (as an internal control). Primers used were as follows: beta-actin, forward 5′-AAGGCCAACC GTGAAAAGAT-3′, reverse 5′-GTGGTACGACCAGAGGCAT AC-3′; Bmp4, forward 5′-GCTGGAATGATTGGATTGTG-3′, reverse 5′-CATGGTTGGTTGAGTTGAGG-3′; Shh, forward 5′-TTCTGTGAAAGCAGAGAACTCC-3′, reverse 5′-GGGAC GTAAGTCCTTCACCA-3′; Tgfb1, forward 5′-TACCATGCC AACTTCTGTCTGGGA-3′, reverse 5′-ATGTTGGACAACTGC TCCACCTTG-3′; Wnt3a, forward 5′-GAACCGTCACAAC AATGAGG-3′, reverse 5′-CTTCACAGCTGCCAGATAGC-3′; Gli1, forward 5′-CCAAGCCAACTTTATGTCAGGG-3′, reverse 5′-AGCCCGCTTCTTTGTTAATTTGA-3′; Ptc1, forward 5′-CG AGACCAACGTGGAGGAGC-3′, reverse 5′-GGAGTCTGTA TCATGAGTTGAGG-3′; Smo, forward 5′-GGAGGGTTCCC AGGGTTGAA-3′, reverse 5′-GCCCCTCGACTCCCAACTT-3′.

### Western Blotting

Cell aggregates treated with or without inhibitors from day 5 were dissociated using TrypLE Express Enzyme (Thermo Fisher Scientific) on day 6 and lysed in radioimmunoprecipitation assay buffer containing 50-mM Tris hydrochloride (pH 7.6), 150-mM sodium chloride, 1% Nonidet P40, 0.5% sodium deoxycholate, and 0.1% sodium dodecyl sulfate with protease inhibitor cocktail (Nacalai) and ethylenediaminetetraacetic acid-free phosphatase inhibitor cocktail (Nacalai). A total of 2.7–9.0 μg of protein extracts were separated by 10% sodium dodecyl sulfate–polyacrylamide gel electrophoresis and blotted onto polyvinylidene fluoride membrane (Merck, Immobilon). Three biological replicates were analyzed. The following primary antibodies were used at the indicated dilutions: anti-beta-actin (rabbit, 1:1,000; Cell Signaling, 4970), anti-beta-catenin (rabbit, 1:1,000; Cell Signaling, 8480), anti-phospho-Smad1/5 (rabbit, 1:1,000; Cell Signaling, 9516), and anti-phospho-Smad2 (rabbit, 1:1,000; Cell Signaling, 3108). Anti-rabbit IgG, HRP-linked antibody (rabbit, 1:2,000; Cell Signaling, 7074) was used as a secondary antibody. The blocking buffer used was Western BLoT Immuno Booster (TaKaRa). The chemiluminescent substrate used was Western BLoT Ultra-Sensitive HRP Substrate (TaKaRa). The chemiluminescent signal was detected using LAS 4000mini (Cytiva) and analyzed using ImageJ.

### Statistical Analyses

All statistical tests were conducted using R. Multiple-group means were compared by one-way ANOVA with *post hoc* Dunnett’s test (Figures 2, 5, 7) or Williams’ tests (Figure 4) for pairwise comparisons or by two-way ANOVA with *post hoc* Dunnett’s test (Figure 6). Frequencies were compared by Fisher’s exact test (Figure 2). A *P* < 0.05 (two-tailed) was considered significant for all tests.

## Data Availability Statement

The original contributions presented in the study are included in the article/Supplementary Material, further inquiries can be directed to the corresponding author/s.

## Ethics Statement

The animal study was reviewed and approved by the Animal Care and Use Committee of Kumamoto University.

## Author Contributions

MN conceived and designed the research, performed all experiments and analyses, and wrote the manuscript. SE, JH, and NT helped to perform the experiments. KS contributed to finalizing the manuscript. All authors contributed to the article and approved the submitted version.

## Conflict of Interest

The authors declare that the research was conducted in the absence of any commercial or financial relationships that could be construed as a potential conflict of interest.

## References

[B1] Aldiri I. Xu B. Wang L. Chen X. Hiler D. Griffiths L. (2017). The dynamic epigenetic landscape of the retina during development, reprogramming, and tumorigenesis. *Neuron* 94 550–568.e10. 10.1016/j.neuron.2017.04.022 28472656 PMC5508517

[B2] Arber C. Precious S. V. Cambray S. Risner-Janiczek J. R. Kelly C. Noakes Z. (2015). Activin A directs striatal projection neuron differentiation of human pluripotent stem cells. *Development* 142 1375–1386. 10.1242/dev.117093 25804741 PMC4378247

[B3] Backman M. Machon O. Mygland L. Van Den Johannes C. J. Zhong W. (2005). Effects of canonical Wnt signaling on dorso-ventral specification of the mouse telencephalon. *Dev. Biol.* 279 155–168. 10.1016/j.ydbio.2004.12.010 15708565

[B4] Bertacchi M. Pandolfini L. D’Onofrio M. Brandi R. Cremisi F. (2015). The double inhibition of endogenously produced bmp and wnt factors synergistically triggers dorsal telencephalic differentiation of mouse es cells. *Dev. Neurobiol.* 75 66–79. 10.1002/dneu.22209 25044881

[B5] Cambray S. Arber C. Little G. Dougalis A. G. de Paola V. Ungless M. A. (2012). Activin induces cortical interneuron identity and differentiation in embryonic stem cell-derived telencephalic neural precursors. *Nat. Commun.* 3:841. 10.1038/ncomms1817 22588303

[B6] Campbell K. (2003). Dorsal-ventral patterning in the mammalian telencephalon. *Curr. Opin. Neurobiol.* 13 50–56. 10.1016/S0959-4388(03)00009-6 12593982

[B7] Chen B. Dodge M. E. Tang W. Lu J. Ma Z. Fan C. (2009). Small molecule-mediated disruption of Wnt-dependent signaling in tissue regeneration and cancer. *Nat. Chem. Biol.* 5 100–107. 10.1038/nchembio.137 19125156 PMC2628455

[B8] Corbin J. G. Gaiano N. Machold R. P. Langston A. Fishell G. (2000). The Gsh2 homeodomain gene controls multiple aspects of telencephalic development. *Development* 127 5007–5020. 11060228 10.1242/dev.127.23.5007

[B9] Corbin J. G. Rutlin M. Gaiano N. Fishell G. (2003). Combinatorial function of the homeodomain proteins Nkx2.1 and Gsh2 in ventral telencephalic patterning. *Development* 130 4895–4906. 10.1242/dev.00717 12930780

[B10] Danjo T. Eiraku M. Muguruma K. Watanabe K. Kawada M. Yanagawa Y. (2011). Subregional specification of embryonic stem cell-derived ventral telencephalic tissues by timed and combinatory treatment with extrinsic signals. *J. Neurosci.* 31 1919–1933. 10.1523/JNEUROSCI.5128-10.2011 21289201 PMC6623725

[B11] Dessaud E. Yang L. L. Hill K. Cox B. Ulloa F. Ribeiro A. (2007). Interpretation of the sonic hedgehog morphogen gradient by a temporal adaptation mechanism. *Nature* 450 717–720. 10.1038/nature06347 18046410

[B12] Eiraku M. Watanabe K. Matsuo-Takasaki M. Kawada M. Yonemura S. Matsumura M. (2008). Self-organized formation of polarized cortical tissues from ESCs and its active manipulation by extrinsic signals. *Cell Stem Cell* 3 519–532. 10.1016/j.stem.2008.09.002 18983967

[B13] Fuccillo M. Rallu M. McMahon A. P. Fishell G. (2004). Temporal requirement for hedgehog signaling in ventral telencephalic patterning. *Development* 131 5031–5040. 10.1242/dev.01349 15371303

[B14] Hébert J. M. Mishina Y. McConnell S. K. (2002). BMP signaling is required locally to pattern the dorsal telencephalic midline. *Neuron* 35 1029–1041. 10.1016/S0896-6273(02)00900-5 12354394

[B15] Hirabayashi Y. Suzki N. Tsuboi M. Endo T. A. Toyoda T. Shinga J. (2009). Polycomb limits the neurogenic competence of neural precursor cells to promote astrogenic fate transition. *Neuron* 63 600–613. 10.1016/j.neuron.2009.08.021 19755104

[B16] Imayoshi I. Shimogori T. Ohtsuka T. Kageyama R. (2008). Hes genes and neurogenin regulate non-neural versus neural fate specification in the dorsal telencephalic midline. *Development* 135 2531–2541. 10.1242/dev.021535 18579678

[B17] Lek M. Dias J. M. Marklund U. Uhde C. W. Kurdija S. Lei Q. (2010). A homeodomain feedback circuit underlies step-function interpretation of a Shh morphogen gradient during ventral neural patterning. *Development* 137 4051–4060. 10.1242/dev.054288 21062862

[B18] Marín O. Rubenstein J. L. R. (2001). A long, remarkable journey: tangential migration in the telencephalon. *Nat. Rev. Neurosci.* 2 780–790. 11715055 10.1038/35097509

[B19] Maroof A. M. Keros S. Tyson J. A. Ying S.-W. Ganat Y. M. Merkle F. T. (2013). Directed differentiation and functional maturation of cortical interneurons from human embryonic stem cells. *Cell Stem Cell* 12 559–572. 10.1016/j.stem.2013.04.008 23642365 PMC3681523

[B20] Medina L. Abellán A. (2009). Development and evolution of the pallium. *Semin. Cell Dev. Biol* 20 698–711. 10.1016/j.semcdb.2009.04.008 19393324

[B21] Métin C. Baudoin J.-P. Rakiæ S. Parnavelas J. G. (2006). Cell and molecular mechanisms involved in the migration of cortical interneurons. *Eur. J. Neurosci.* 23 894–900. 10.1111/j.1460-9568.2006.04630.x 16519654

[B22] Moore S. Ribes V. Terriente J. Wilkinson D. Relaix F. Briscoe J. (2013). Distinct regulatory mechanisms act to establish and maintain pax3 expression in the developing neural tube. *PLoS Genet.* 9:e1003811. 10.1371/journal.pgen.1003811 24098141 PMC3789833

[B23] Morikawa M. Koinuma D. Mizutani A. Kawasaki N. Holmborn K. Sundqvist A. (2016). BMP sustains embryonic stem cell self-renewal through distinct functions of different krüppel-like factors. *Stem Cell Reports* 6 64–73. 10.1016/j.stemcr.2015.12.004 26771354 PMC4719190

[B24] Nasu M. Shimamura K. Esumi S. Tamamaki N. (2020a). Sequential pattern of sublayer formation in the paleocortex and neocortex. *Med. Mol. Morphol.* 53 168–176. 10.1007/s00795-020-00245-7 32002665

[B25] Nasu M. Shimamura K. Esumi S. Tamamaki N. (2020b). Formation of dorsal–ventral axis of the pallium derived from mouse embryonic stem cells. *Biochem. Biophys. Res. Commun.* 524 117–122. 10.1016/j.bbrc.2020.01.070 31980168

[B26] Nasu M. Takata N. Danjo T. Sakaguchi H. Kadoshima T. Futaki S. (2012). Robust formation and maintenance of continuous stratified cortical neuroepithelium by laminin-containing matrix in mouse ES cell culture. *PLoS One* 7:e53024. 10.1371/journal.pone.0053024 23300850 PMC3534089

[B27] Naujok O. Lentes J. Diekmann U. Davenport C. Lenzen S. (2014). Cytotoxicity and activation of the Wnt/beta-catenin pathway in mouse embryonic stem cells treated with four GSK3 inhibitors. *BMC Res. Notes* 7:273. 10.1186/1756-0500-7-273 24779365 PMC4008422

[B28] Nery S. Wichterle H. Fishell G. (2001). Sonic hedgehog contributes to oligodendrocyte specification in the mammalian forebrain. *Development* 128 527–540. 11171336 10.1242/dev.128.4.527

[B29] Rallu M. Corbin J. G. Fishell G. (2002a). Parsing the prosencephalon. *Nat. Rev. Neurosci.* 3 943–951. 10.1038/nrn989 12461551

[B30] Rallu M. Machold R. Gaiano N. Corbin J. G. Mcmahon A. P. (2002b). Dorsoventral patterning is established in the telencephalon of mutants lacking both Gli3 and Hedgehog signaling. *Development* 129 4963–4974. 12397105 10.1242/dev.129.21.4963

[B31] Ribes V. Briscoe J. (2009). Establishing and interpreting graded Sonic Hedgehog signaling during vertebrate neural tube patterning: the role of negative feedback. *Cold Spring Harb. Perspect. Biol.* 1:a002014. 10.1101/cshperspect.a002014 20066087 PMC2742090

[B32] Rogers K. W. Schier A. F. (2011). Morphogen gradients: from generation to interpretation. *Annu. Rev. Cell Dev. Biol.* 27 377–407. 10.1146/annurev-cellbio-092910-154148 21801015

[B33] Sagner A. Briscoe J. (2017). Morphogen interpretation: concentration, time, competence, and signaling dynamics. *Wiley Interdiscip. Rev. Dev. Biol.* 6:e271. 10.1002/wdev.271 28319331 PMC5516147

[B34] Sasai N. Kutejova E. Briscoe J. (2014). Integration of signals along orthogonal axes of the vertebrate neural tube controls progenitor competence and increases cell diversity. *PLoS Biol.* 12:e1001907. 10.1371/journal.pbio.1001907 25026549 PMC4098999

[B35] Schuurmans C. Guillemot F. (2002). Molecular mechanisms underlying cell fate specification in the developing telencephalon. *Curr. Opin. Neurobiol.* 12 26–34. 10.1016/S0959-4388(02)00286-6 11861161

[B36] Shimamura K. Hartigan D. J. Martinez S. Puelles L. Rubenstein J. L. (1995). Longitudinal organization of the anterior neural plate and neural tube. *Development* 121 3923–3933. 8575293 10.1242/dev.121.12.3923

[B37] Shindo Y. Iwamoto K. Mouri K. Hibino K. Tomita M. Kosako H. (2016). Conversion of graded phosphorylation into switch-like nuclear translocation via autoregulatory mechanisms in ERK signalling. *Nat. Commun.* 7:10485. 10.1038/ncomms10485 26786866 PMC4736105

[B38] Sousa V. H. Fishell G. (2010). Sonic hedgehog functions through dynamic changes in temporal competence in the developing forebrain. *Curr. Opin. Genet. Dev.* 20 391–399. 10.1016/j.gde.2010.04.008 20466536 PMC2991106

[B39] Srinivasan S. Hu J. S. Currle D. S. Fung E. S. Hayes W. B. Lander A. D. (2014). A BMP-FGF morphogen toggle switch drives the ultrasensitive expression of multiple genes in the developing forebrain. *PLoS Comput. Biol.* 10:e1003463. 10.1371/journal.pcbi.1003463 24550718 PMC3923663

[B40] Subramanian L. Tole S. (2009). Mechanisms underlying the specification, positional regulation, and function of the cortical hem. *Cereb. Cortex.* 19 90–95. 10.1093/cercor/bhp031 19359348

[B41] Sussel L. Marin O. Kimura S. Rubenstein J. L. R. (1999). Loss of Nkx2. 1 homeobox gene function results in a ventral to dorsal molecular respecification within the basal telencephalon: evidence for a transformation of the pallidum into the striatum. *Development* 126 3359–3370. 10393115 10.1242/dev.126.15.3359

[B42] Tamamaki N. Fujimori K. E. Takauji R. (1997). Origin and route of tangentially migrating neurons in the developing neocortical intermediate zone. *J. Neurosci.* 17 8313–8323. 9334406 10.1523/JNEUROSCI.17-21-08313.1997 PMC6573720

[B43] Thomas T. Dziadek M. (1993). Capacity to form choroid plexus-like cells in vitro is restricted to specific regions of the mouse neural ectoderm. *Development* 117 253–262. 8223250 10.1242/dev.117.1.253

[B44] Toresson H. Potter S. S. Campbell K. (2000). Genetic control of dorsal-ventral identity in the telencephalon: opposing roles for Pax6 and Gsh2. 127 4361–4371. 10.1242/dev.127.20.4361 11003836

[B45] Tyson J. A. Goldberg E. M. Maroof A. M. Xu Q. Petros T. J. Anderson S. A. (2015). Duration of culture and sonic hedgehog signaling differentially specify PV versus SST cortical interneuron fates from embryonic stem cells. *Development* 142 1267–1278. 10.1242/dev.111526 25804737 PMC4378243

[B46] van Boxtel A. L. Chesebro J. E. Heliot C. Ramel M. C. Stone R. K. Hill C. S. (2015). A temporal window for signal activation dictates the dimensions of a nodal signaling domain. *Dev. Cell.* 35 175–185. 10.1016/j.devcel.2015.09.014 26506307 PMC4640439

[B47] Watanabe K. Kamiya D. Nishiyama A. Katayama T. Nozaki S. Kawasaki H. (2005). Directed differentiation of telencephalic precursors from embryonic stem cells. *Nat. Neurosci.* 8 288–296. 10.1038/nn1402 15696161

[B48] Watanabe M. Kang Y.-J. Davies L. M. Meghpara S. Lau K. Chung C.-Y. (2012). BMP4 sufficiency to induce choroid plexus epithelial fate from embryonic stem cell-derived neuroepithelial progenitors. *J. Neurosci.* 32 15934–15945. 10.1523/JNEUROSCI.3227-12.2012 23136431 PMC3505486

[B49] Wilson S. W. Rubenstein J. L. R. (2000). Induction and dorsoventral patterning of the telencephalon. *Neuron* 28 641–651. 10.1016/S0896-6273(00)00171-9 11163256

[B50] Wolpert L. (1969). Positional information and the spatial pattern of cellular differentiation. *J. Theor. Biol.* 25 1–47. 10.1016/S0022-5193(69)80016-0 4390734

[B51] Wonders C. P. Anderson S. A. (2006). The origin and specification of cortical interneurons. *Nat. Rev. Neurosci.* 7 687–696. 10.1038/nrn1954 16883309

[B52] Wu S. Esumi S. Watanabe K. Chen J. Nakamura K. C. Nakamura K. (2011). Tangential migration and proliferation of intermediate progenitors of GABAergic neurons in the mouse telencephalon. *Development* 138 2499–2509. 10.1242/dev.063032 21561989

[B53] Xu Q. Guo L. Moore H. Waclaw R. R. Campbell K. Anderson S. A. (2010). Sonic hedgehog signaling confers ventral telencephalic progenitors with distinct cortical interneuron fates. *Neuron* 65 328–340. 10.1016/j.neuron.2010.01.004 20159447 PMC2868511

[B54] Xu Q. Tam M. Anderson S. A. (2008). Fate mapping Nkx2.1-lineage cells in the mouse telencephalon. *J. Comp. Neurol.* 506 16–29. 10.1002/cne.21529 17990269

[B55] Xu Q. Wonders C. P. Anderson S. A. (2005). Sonic hedgehog maintains the identity of cortical interneuron progenitors in the ventral telencephalon. *Development* 132 4987–4998. 10.1242/dev.02090 16221724

[B56] Yun K. Potter S. Rubenstein J. L. R. (2001). Gsh2 and Pax6 play complementary roles in dorsoventral patterning of the mammalian telencephalon. *Development* 128 193–205. 11124115 10.1242/dev.128.2.193

[B57] Zhang Q. Andersen M. E. Conolly R. B. (2006). Binary gene induction and protein expression in individual cells. *Theor. Biol. Med. Model.* 3:18. 10.1186/1742-4682-3-18 16597340 PMC1488830

