# Supplementary material for: Two-Phase Lineage Specification of Telencephalon Progenitors Generated From Mouse Embryonic Stem Cells
Source: Front Cell Dev Biol. 2021 Apr 16;9:632381. doi: 10.3389/fcell.2021.632381 (PMC8086603; doi:10.3389/fcell.2021.632381)
Supplement: Supplementary file 1 [file Data_Sheet_1.docx]

Supplementary Material

# Supplementary Figures


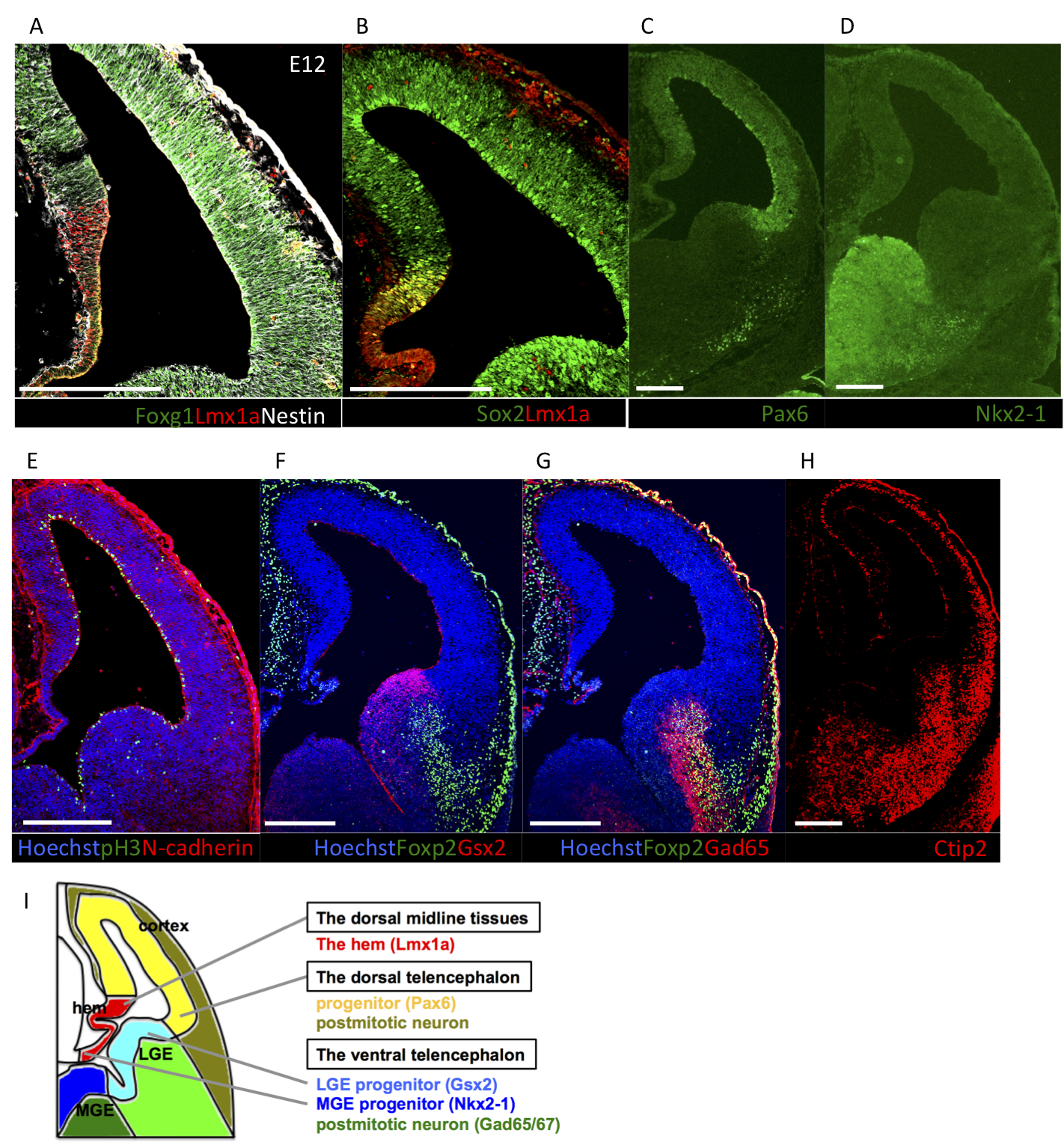


**Supplementary Figure 1.** *In vivo* expression pattern of the region-specific markers used to determine lineage induction**. (A–H)** Expression of marker genes in E12 mouse telencephalon: **(A)** Foxg1 (pan-telencephalon, green), Lmx1a (dorsal midline tissues, red) and Nestin (neural lineage-committed cells; white); **(B)** Sox2 (neural progenitor cells, green) and Lmx1a (red); **(C)** Pax6 (pallium or cortex, green); **(D)** Nkx2.1 (MGE/POA, green); **(E)** pH3 (mitotic cells, green) and N-cadherin (adherens junction, red); **(F)** Foxp2 (LGE/CGE neuron, green) and Gsx2 (LGE/CGE, red); **(G)** Foxp2 (green) and Gad65 (pan-GE neuron and intermediate progenitor, red); **(H)** Ctip2 (pan-GE neuron and a part of cortical neurons, red). Hoechst 33342 (blue), nuclear staining. Scale bars, 500 µm. **(I)** Schematic of nested expression of sub-region markers in the whole telencephalon.


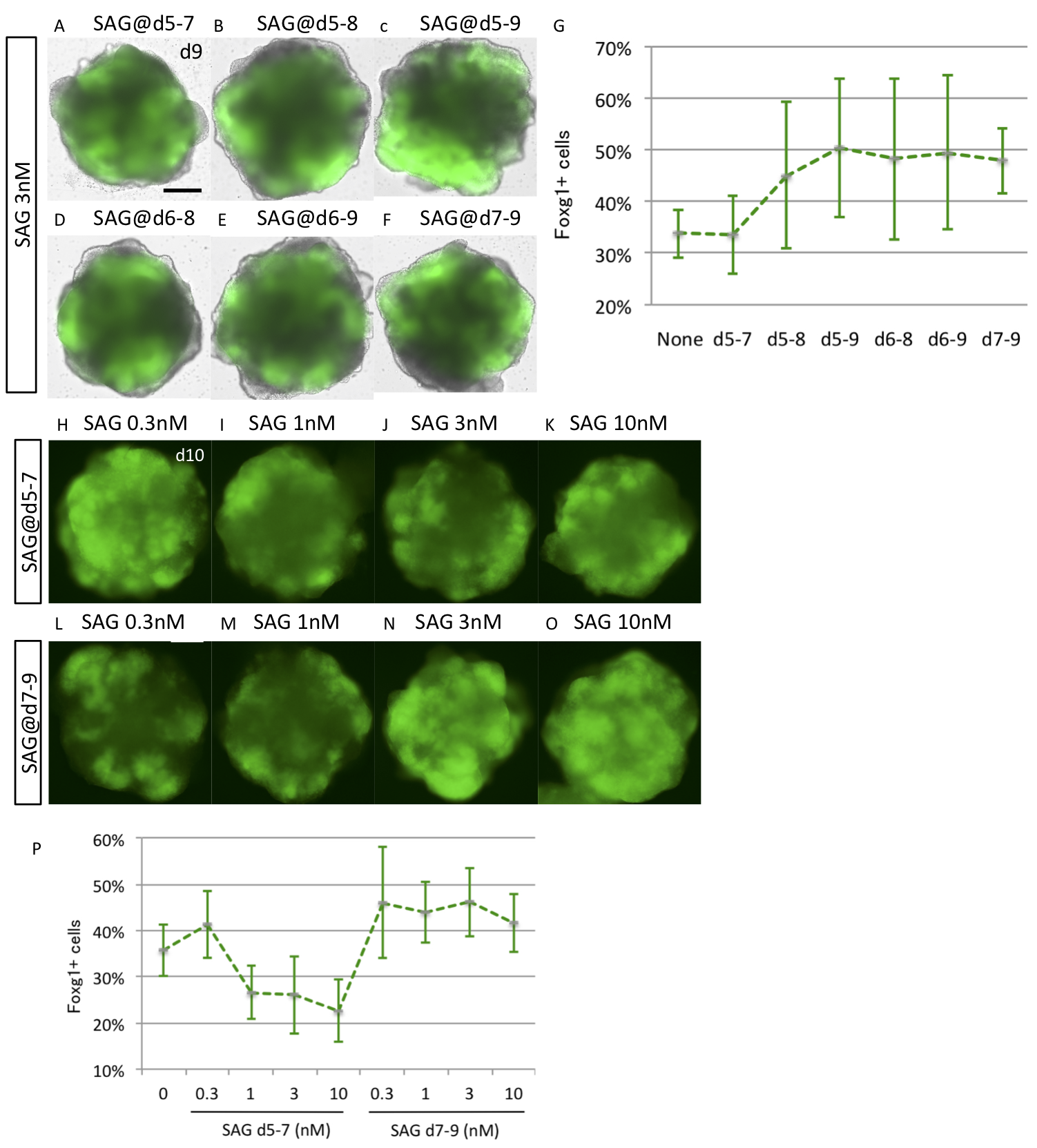


**Supplementary Figure 2.** Whole-mount fluorescence images of Foxg1::venus aggregates, corresponding to Figures 2, 4. **(A–F)** Whole-mount fluorescence images on day 9 merged with differential interference contrast (DIC) images, corresponding to Figure 2. **(G)** The proportion (%) of Foxg1^+^ cells in aggregates cultured with 3 nM SAG for different durations and at different onset times. A large proportion of cultured cells differentiated into Foxg1^+^ telencephalon progenitors independent of the timing of SAG exposure. Values expressed as mean ± SEM (N = 4). **(H–O)** Whole-mount fluorescence images on day 10, corresponding to Figure 4. **(P)** A large proportion of cultured cells differentiated into Foxg1^+^ telencephalon progenitors independent of SAG concentration. Values expressed as mean ± SEM (N = 3). Scale bars, 200 µm.


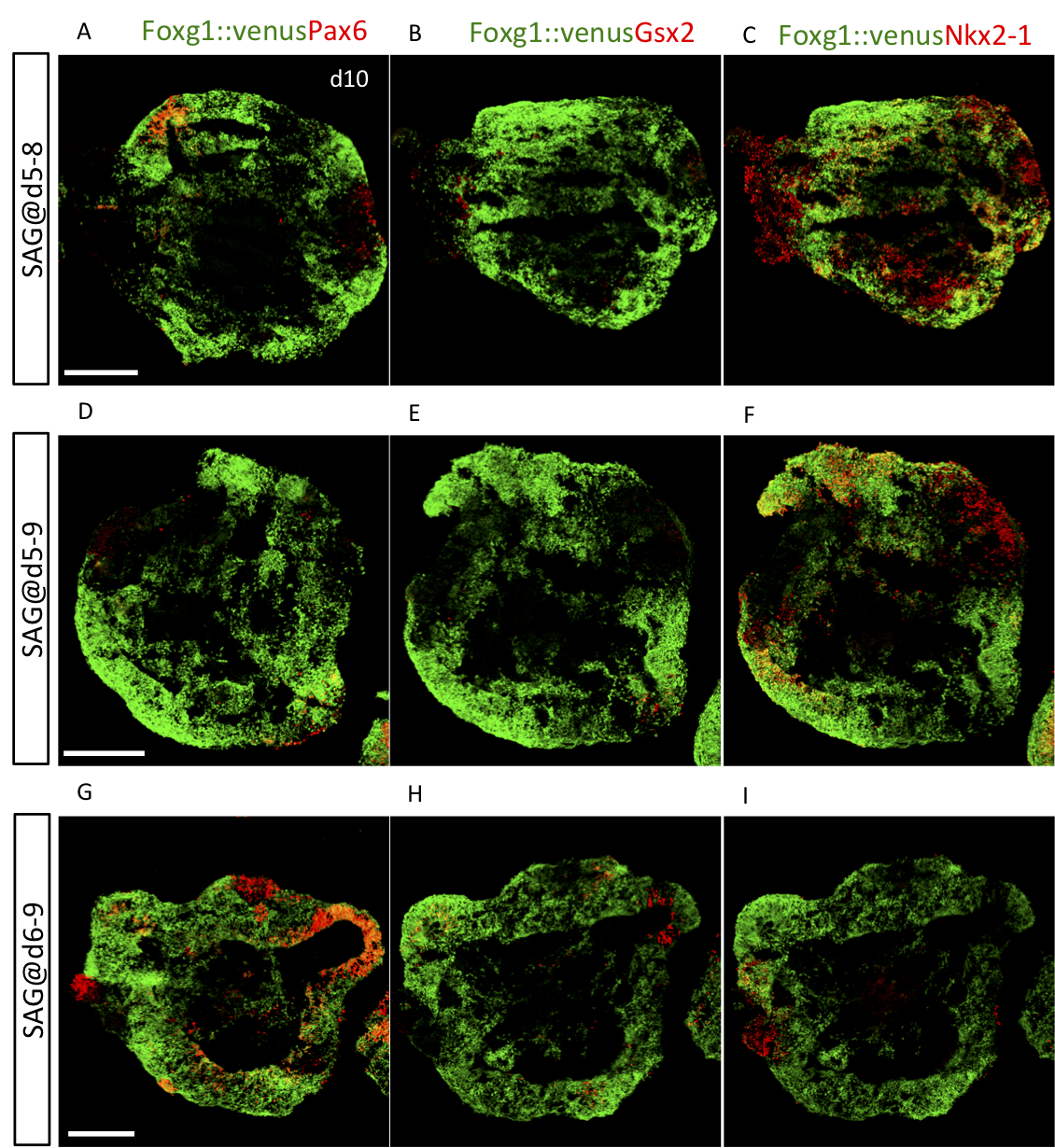


**Supplementary Figure 3.** Aggregates cultured with different durations and timing of SAG. Fluorescence images of serial sections, corresponding to Figure 2. **(A–I)** Foxg1::venus (telencephalic) cell aggregates (green) co-immunostained for sub-regional markers (red): Pax6 (A,D,G), Gsx2 (B,E,H) and Nkx2-1 (C,F,I). Aggregates cultured with 3 nM SAG during days 5–8 (A–C) and days 5–9 (D–F) expressed primarily Nkx2-1. Aggregates cultured with 3 nM SAG during days 6–9 expressed Pax6, Gsx2 and Nkx2-1 (G–I). Scale bars, 200 µm.


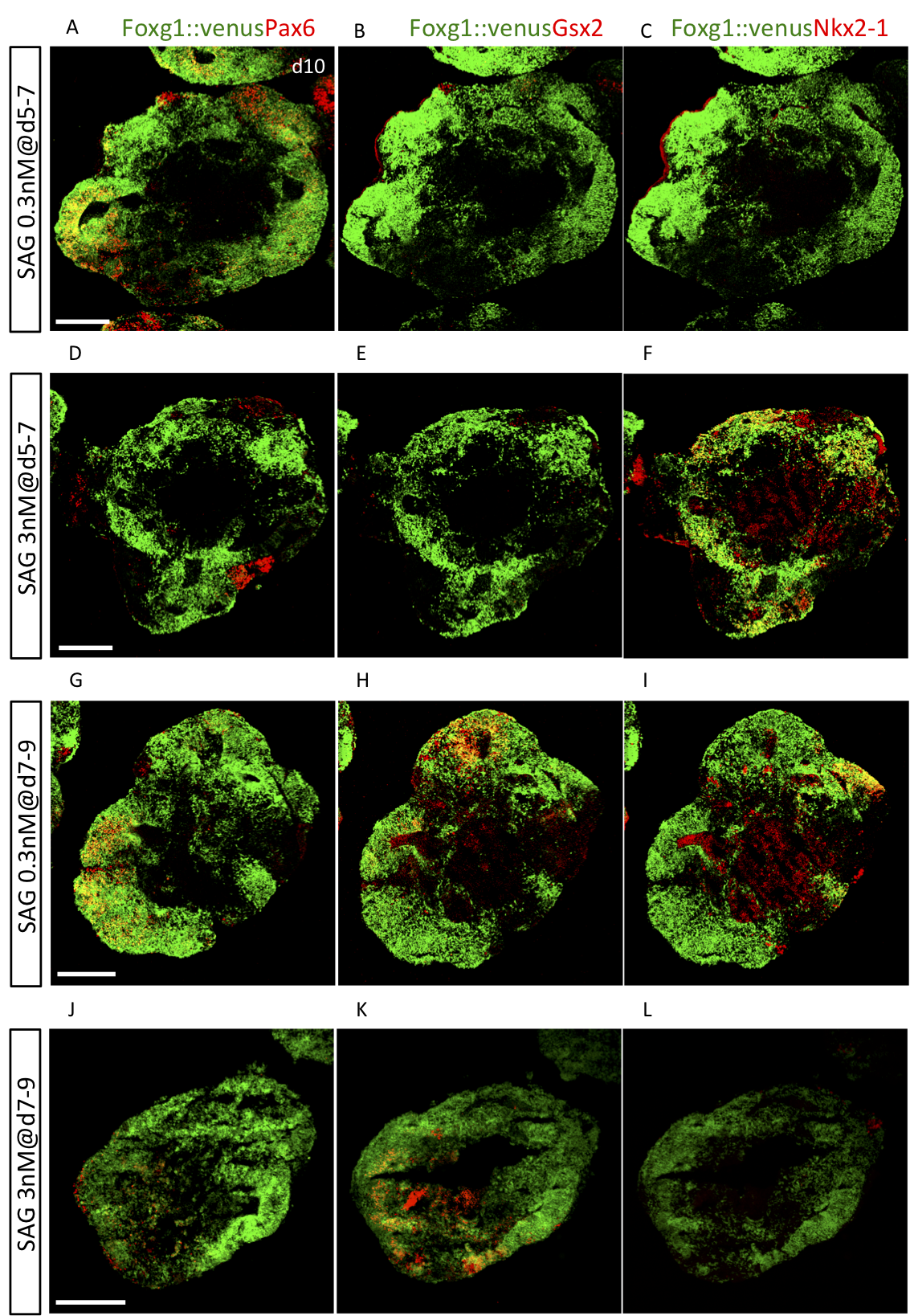


**Supplementary Figure 4.** Aggregates cultured with different concentrations of SAG during the early phase. Fluorescence images of serial sections, corresponding to Figure 4. **(A–F)** Aggregates cultured with 0.3 or 3 nM SAG during the early phase (days 5–7). **(G–L)** Aggregates cultured with 0.3 or 3 nM SAG during the late phase (days 7–9). Foxg1::venus (telencephalic) cell aggregates (green) co-immunostained for sub-regional markers (red): Pax6 (A,D,G,J), Gsx2 (B,E,H,K) and Nkx2-1 (C,F,I,L). Scale bars, 200 µm.


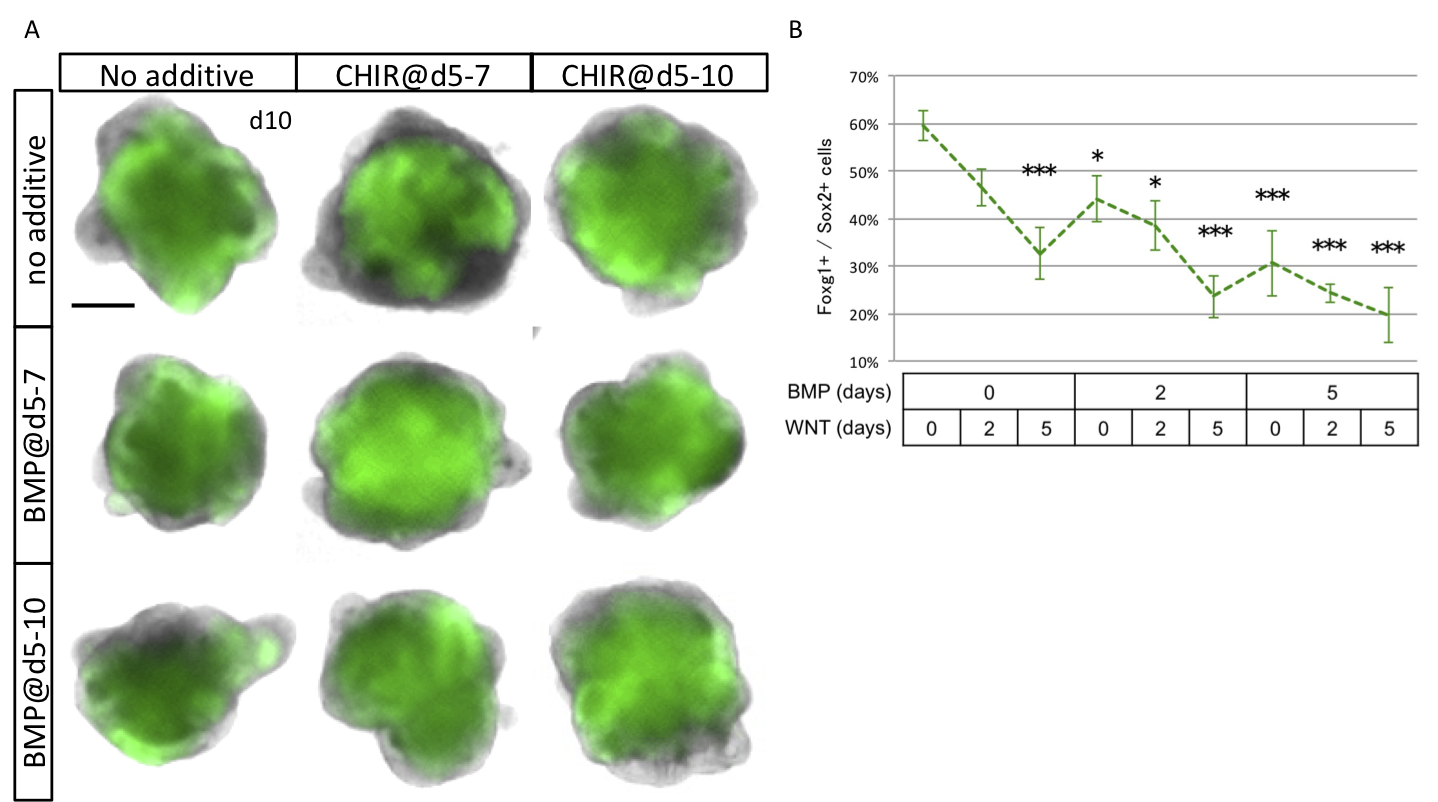


**Supplementary Figure 5.** Whole-mount fluorescence images of Foxg1::venus aggregates cultured with different combinations of BMP4 and CHIR99021, corresponding to Figure 6. **(A)** Whole-mount fluorescence images of Foxg1::venus aggregates on day 10 merged with DIC images. Scale bars, 200 µm. **(B)** The proportion (%) of Foxg1^+^ cells among Sox2^+^ cells cultured with BMP4 and/or CHIR99021 for 2 and 5 days starting on day 5. Values represent the mean ± SEM. *P < 0.05, ***P < 0.001.


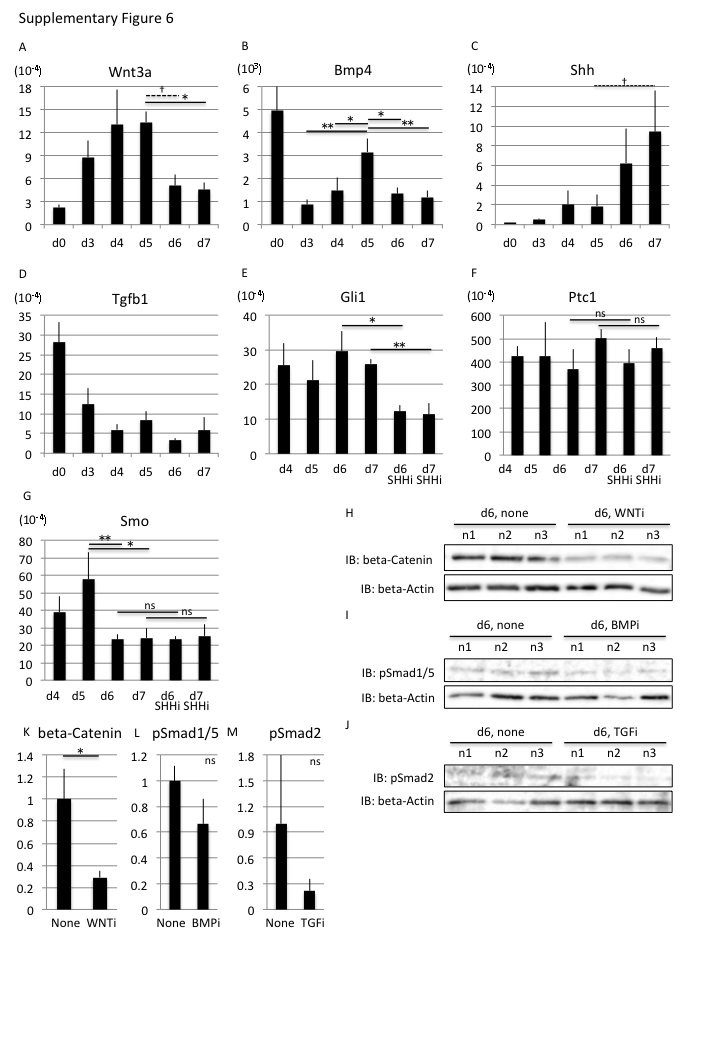


**Supplementary Figure 6.** Qunatitative study of RNA and protein. **(A–G)** qPCR study. Beta-Actin as an internal control. **(A–D)** Relative expressions of Wnt3a (A), Bmp4 (B), Shh (C) and Tgfb1 (D). ES cells under maintenance culture (d0) and differentiating cells from day 3 (d3) to day 7 (d7) were analyzed. Multiple-group means during days 3–7 were compared by Dunnett's multiple-comparisons with day 5. **(E–G)** Relative expressions of genes in the Shh signaling pathway; Gli1 (E), Ptc1 (F) and Smo (G). Differentiating cells from day 4 (d4) to day 7 (d7) were analyzed. SHHi was added during days 5–7. Multiple-group means during days 4–7 were compared by Dunnett's multiple-comparisons with day 5. Pairwise comparisons among day6 or day 7 were made using Student's t-test. **(H–J)** Western blotting of protein extracts from three biological replicates (n1, n2, n3) on day 6. Beta-Actin as an internal control (IB: beta-Actin). **(H)** Western blotting for beta-Catenin (IB: beta-Catenin) after WNTi treatment from day5 (d6, WNTi) or none (d6, none). **(I)** Western blotting for phospho-Smad1/5 (IB: pSmad1/5) after BMPi treatment from day5 (d6, BMPi) or none (d6, none). **(J)** Western blotting for phospho-Smad2 (IB: pSmad2) after TGFi treatment from day5 (d6, TGFi) or none (d6, none). **(K–M)** Relative values of fluorescent intensities of western blotting, based on values of beta-Actin. Value of the group with no inhibitor was set to 1. Pairwise comparisons were made using Student's t-test. Values represent the mean ± SEM (N = 3). *P < 0.05, **P < 0.01, †P < 0.1. ns, not significant.


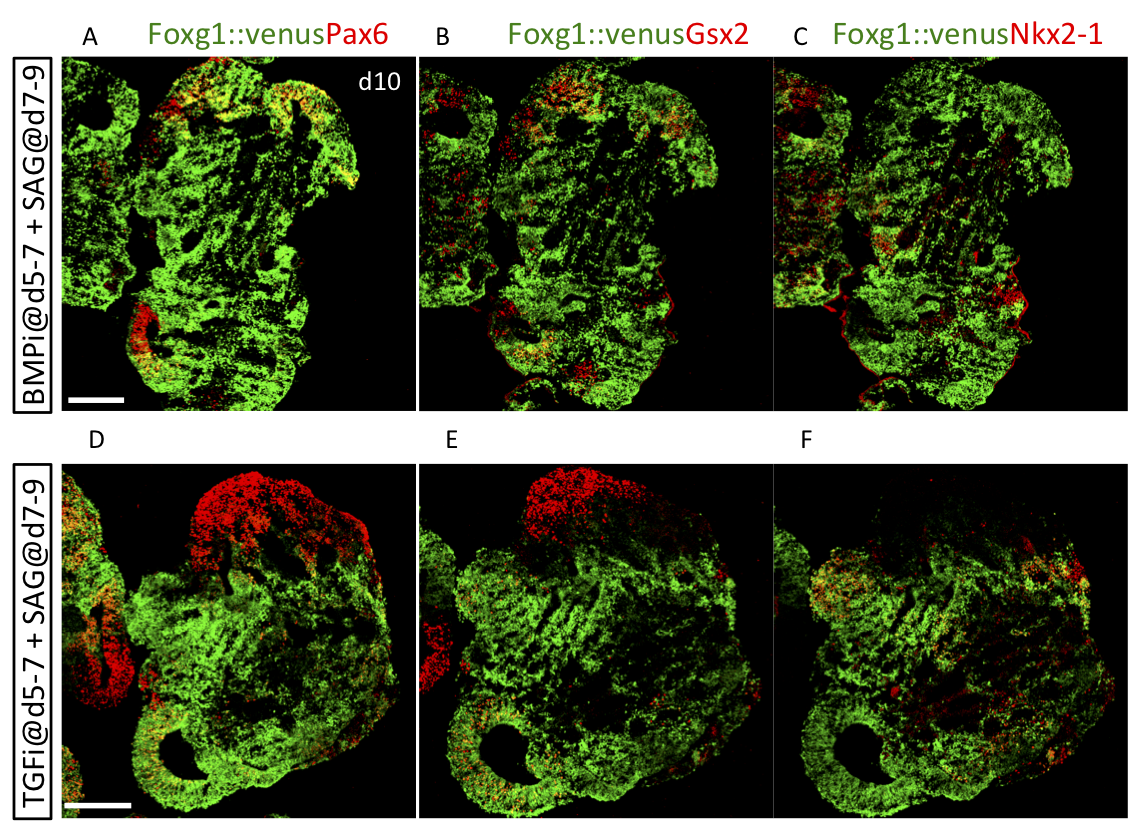


**Supplementary Figure 7.** Aggregates cultured with pre-treatment of inhibitors and late exposure to SAG. Fluorescence images of serial sections, corresponding to Figure 7. **(A–F)** Foxg1::venus (telencephalic) cell aggregates (green) co-immunostained for sub-regional markers (red): Pax6 (A,D), Gsx2 (B,E) and Nkx2-1 (C,F). Aggregates cultured with inhibitors for the BMP (A–C) and TGFb (D–F) signaling pathway during days 5–7 and then treated with SAG during days 7–9. Scale bars, 200 µm.
